# Polytetrafluoroethylene utensils as a source of micro- and nanoplastics: Insights from a *Drosophila* model

**DOI:** 10.1016/j.eehl.2026.100285

**Published:** 2026-09-15

**Authors:** Mohamed Alaraby, Doaa Abass, Elham Farghal Elkady, Raquel Egea, Antonia Velázquez, Alba Hernández, Ricard Marcos

**Affiliations:** a Group of Mutagenesis, Department of Genetics and Microbiology, Faculty of Biosciences, Universitat Autònoma de Barcelona, Cerdanyola del Vallès, 08193, Spain; b Zoology Department, Faculty of Sciences, Sohag University, Sohag, 82524, Egypt; c Plant Protection, Institute of Agricultural Research Center, Sohag, 82524, Egypt

**Keywords:** Polytetrafluoroethylene (PTFE), Micro- and nanoplastics (MNPLs), Uptake dynamics, Mitochondrial toxicity, Metabolism, Transcriptome, Oxidative stress, Genotoxicity

## Abstract

Polytetrafluoroethylene (PTFE), widely used in household products and cookware, can release particles into food, posing potential health risks. The biological interactions of the resulting micro- and nanoplastics (MNPLs) remain poorly understood but can be clarified by linking structural and physiological effects with transcriptomic changes. Here, *Drosophila* was used as an *in vivo* model to assess size-dependent responses to PTFE micro- (MPLs) and nanoscale (NPLs) particles. Following oral exposure, PTFE-MNPLs accumulated in the midgut lumen and interacted with gut components, leading to particle erosion, fragmentation, and the release of secondary ultrafine particles. Internalised particles adhered to and damaged gut bacteria, penetrated the intestinal barrier, and compromised epithelial integrity by impairing microvilli, lysosomes, and mitochondria, ultimately entering circulation and interacting with haemocytes. Associated physiological disturbances, including oxidative stress, mitochondrial membrane depolarization, and DNA damage, were reflected in transcriptomic and RT–qPCR analyses, revealing coordinated molecular alterations underlying the observed tissue damage. Analysis also revealed extensive size- and concentration-dependent gene expression changes, with up to 2897 differentially expressed genes for NPLs and a core set of 110 genes consistently affected across treatments, highlighting the common molecular impact of PTFE-MNPL exposure. These genes were enriched in pathways governing metabolism, mitochondrial function, redox balance, immunity, neurodegeneration, apoptosis, intracellular trafficking, and cell migration. Our findings provide a comprehensive view of MNPLs’ biological interactions, linking their physical properties to physiological effects across multiple levels and supported by transcriptomic analysis, particularly for the poorly studied PTFE particles.

## Highlights

• PTFE-MNPL journey after *Drosopila* ingestion is followed using different tools.
• PTFE-MNPLs ingestion triggers transcriptional changes related to immune pathways.
• Inside enterocytes, MNPLs induce morphological and physiological disruptions.
• Lysosomes and mitochondria are primary targets of induced cellular stress.
• Transcriptomics show PTFE-MNPL as modulators of cellular/organismal homeostasis.

## Graphical abstract

**Figure:**
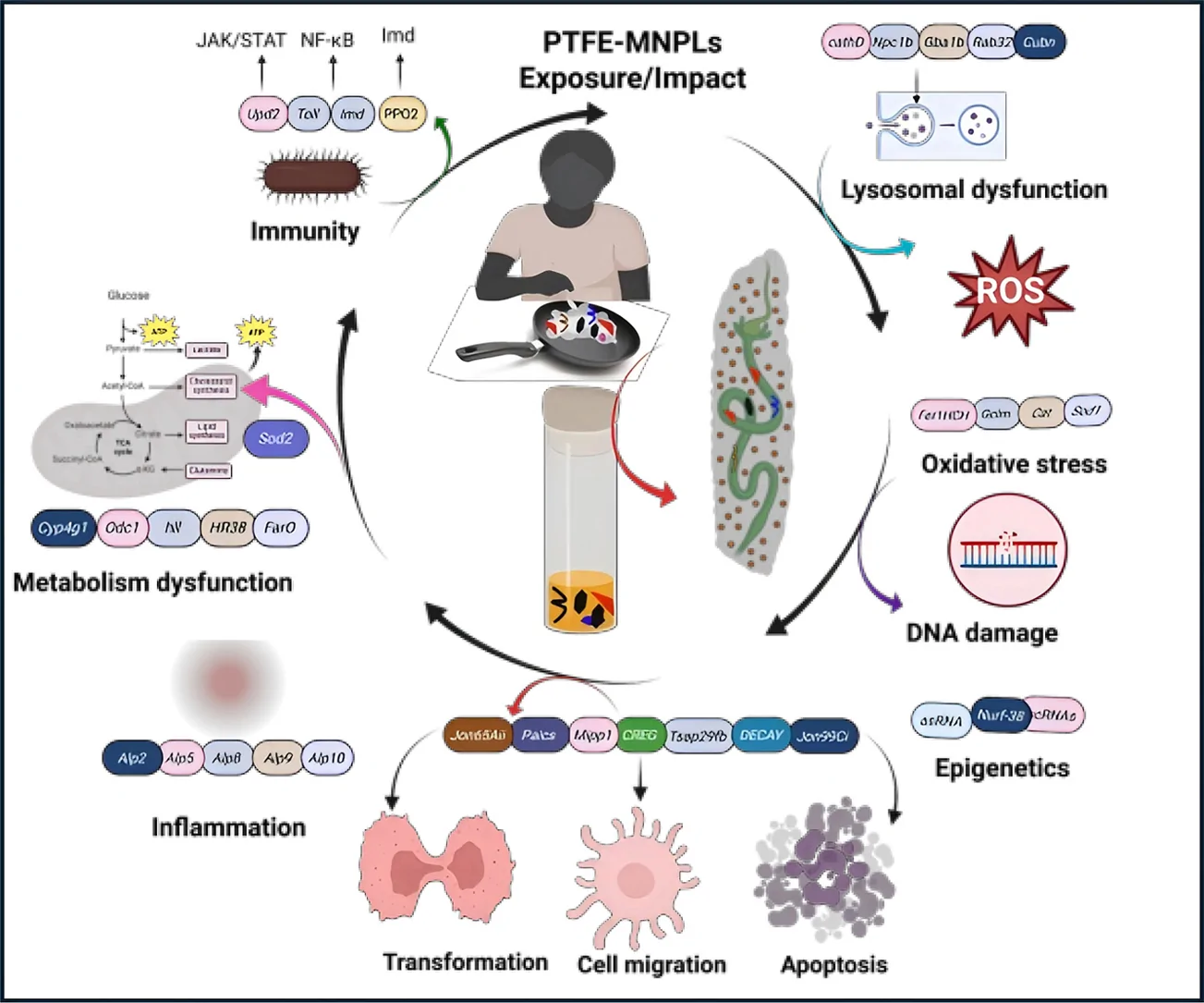

## 1 Introduction

Plastic pollution, with over 359 million tons produced annually and 150–200 million tons accumulating in the environment, represents an unprecedented threat to ecosystem stability and human health [1]. Of particular concern are microplastics and nanoplastics (MNPLs), the ultimate degradation products of larger plastics, which have been detected in human organs, including the kidneys, liver, and brain, thereby raising serious concerns regarding systemic toxicity and long-term exposure risks [[2], [3], [4]]. Exposure to MNPLs occurs via both indirect and direct pathways. Indirect exposure arises through trophic transfer through the food web, with particles bioaccumulating in primary producers and lower-trophic organisms before reaching humans. Direct exposure results from daily use of plastic-containing products, including food packaging, textiles, and household goods [5]. Many approaches have been used to estimate human exposure levels to MNPLs. Estimates suggest that daily human exposure to MNPLs ranges from roughly 0.0002 to 1,531,524 particles per day from diverse sources [6], while independent measures suggest that this exposure correspond to approximately 14–714 mg per day (0.1–5 g per week) [7]. Interestingly, kitchens are a major epicentre of MNPL pollution, as the use of plastic utensils can release substantial quantities of MNPL particles [8]. However, the biological fate of these particles, their interactions with body barriers, and the resulting physiological responses remain poorly understood.

Among the spectrum of plastic pollutants, polytetrafluoroethylene (PTFE) represents a particularly understudied yet pervasive source. Distinguished by its exceptional thermal and chemical resistance, PTFE is extensively utilized in high-performance sectors such as aerospace applications, biomedical devices, and filtration technologies, and is most notably employed as a nonstick coating in cookware [9]. PTFE-based cookware and utensils are now so widespread that they are found in nearly every modern household kitchen [10]. Although PTFE was long regarded as a safe, inert, and insoluble polymer, recent studies have revealed that millions of MNPLs can be released during routine cooking and washing of PTFE-coated utensils, leading to contamination of food, water, and indoor environments [11,12]. PTFE-derived MNPLs have been identified in air, sediments, agricultural soils, and even remote environments, with persistent accumulation reported in shoreline deposits and archaeological layers. Owing to their high density (approximately 2.2 g/cm^3^), PTFE particles readily sediment and are mainly ingested by benthic organisms. In Da Nang City, Vietnam, PTFE was detected across different marine compartments, accounting for 26.5% of the particles identified in seawater and 44.4% in sediments collected from five sampling sites. Its occurrence in marine organisms ranged from 2.6% to 26.9%, with crabs showing the highest PTFE levels [13]. Alarmingly, PTFE particles have also been detected in different human tissues, including lungs, blood, placenta, urine, and reproductive organs [14]. Meta-analyses indicate the presence of PTFE particles across environmental and biological matrices, averaging 7.3 particles/L in water, 3686 particles/kg in sediment, 25 particles/individual in fish, and 483 particles/kg in human tissues [9]. These findings underscore the urgent need for systematic investigations into PTFE particle uptake, internalisation dynamics, and biodegradation, as well as on the cellular mechanisms maintaining homeostasis and the molecular pathways mediating disease.

To elucidate the biological impacts of PTFE-derived MNPLs, we conducted an integrated toxicological assessment using *Drosophila melanogaster* as a model organism. Our study differentiates two PTFE particle types: (i) irregularly shaped MPLs representing environmentally weathered debris (PTFE-MPLs) and (ii) spherical nanoscale particles with uniform morphology (PTFE-NPLs). We systematically characterised their internalisation dynamics, interactions with gut microbiota, translocation across intestinal and systemic barriers, and overall biodistribution. Additionally, we examined physiological, molecular, and genotoxic responses at both organismal and transcriptomic levels, revealing distinct gene networks and stress-response pathways activated upon exposure. This work provides critical mechanistic insights into the fate of PTFE particles, their potential to compromise gut integrity, and their broader impacts on systemic health. By mapping particle behaviour from ingestion through cellular uptake to transcriptional perturbation, we provide a framework for understanding potential human health risks that may be associated with PTFE particles, which could be released from nonstick cookware commonly used in everyday food preparation.

## 2 Materials and methods

### 2.1 Materials

Two distinct size classes of PTFE particles were employed to investigate size-dependent biological effects: micro-sized plastic particles (PTFE-MPLs; heterogeneous, with a broad size distribution) obtained from Nanoshel (UK) and nano-sized plastic particles (PTFE-NPLs; monodisperse) from Sigma-Aldrich. The primary aim of this study was to examine how particle size, a critical physical property, influences biological interactions, internalisation dynamics, and the resulting physiological and molecular responses. To ensure accurate physicochemical profiling, particle morphology, size, shape, and dispersion stability were assessed by scanning electron microscopy (SEM; Zeiss Merlin, Germany), transmission electron microscopy (TEM; HITACHI H-7000, 125 kV, Japan), and dynamic light scattering using a Malvern Zetasizer Nano-ZS (ZEN3600, UK). FTIR spectroscopy (Hyperion 2000, Bruker) was applied to confirm the fluoropolymeric identity of both PTFE types. Stock suspensions (15 mg/mL) were prepared in ultrapure water, sonicated for 16 min (10% amplitude, 400 W probe sonicator) in an ice-water bath, and stirred at 800 rpm for 2 h to prevent particle sedimentation. Suspensions were aliquoted and stored at −80 °C. To enable *in vivo* tracking using confocal microscopy, fluorescently labelled particles were used. For that, PTFE particles (5 mg/mL) were incubated with iDye PolyPink (10 mg/mL) at a 2:1 dye-to-particle ratio, vortexed intermittently for 2 h at a constant temperature, and purified through sequential ultrafiltration using Amicon® Ultra-15 filters (MWCO 100 kDa). Labelled particles were stored at 4 °C protected from light until use.

### 2.2 Exposure

The Canton-S strain of *Drosophila melanogaster* was used to assess developmental and systemic responses to PTFE particle ingestion. PTFE-MNPL suspensions were incorporated into food media at concentrations of 50, 200, and 800 μg/mL, corresponding to 31.25, 125, and 500 μg/g wet food, respectively. Estimated particle numbers were 4.3 × 10^7^, 1.7 × 10^8^, and 6.9 × 10^8^ particles/g food for PTFE-MPLs, and 1.8 × 10^9^, 7.1 × 10^9^, and 2.85 × 10^10^ particles/g food for PTFE-NPLs, respectively, calculated using previously described methods [15]. Considering multiple sources of human MNPL exposure [6,7] and their accumulation over time, even a brief mimic cooking (dry-run, 30 s) can release substantial numbers of PTFE particles from a broken coating area (around 2.3 million), with individual scratches releasing about 9100 particles [11]. Graded PTFE-MNPL concentrations were applied in experimental models to reflect potential human-relevant exposure, based on previously reported ranges, while acknowledging that actual human exposure remains uncertain [[16], [17], [18]]. To evaluate PTFE particle toxicity, fifty eggs were seeded into each vial (five replicates), incubated under standard conditions (24 ± 0.5 °C; 65% ± 5% relative humidity; 12 h:12 h light/dark cycle), and monitored for developmental success and morphological phenotypes. Larvae were exposed for 5 days on 4 g of Drosophila instant medium (Carolina Biological Supply Co.) hydrated with 10 mL of treatment solutions in Milli-Q water at the indicated concentrations, ensuring continuous exposure primarily via ingestion [19]. Exposure was static, with no medium renewal, and larvae were maintained under standard culture conditions without additional handling stress. Concentrations selected for downstream assays were determined based on survival thresholds. Samples (eggs/larvae) were randomly assigned to experimental groups. Imaging and data analyses, including flow cytometry gating where applicable, were performed using predefined, standardized criteria applied consistently across all groups to minimize bias.

### 2.3 Uptake

The internalisation trajectory of both PTFE-MPLs and PTFE-NPLs, from oral exposure to distribution within larval tissues and haemolymph, was tracked using TEM and confocal microscopy as follows:

(i) Transmission Electron Microscopy

Third-instar larvae, five days post-hatching and previously exposed to PTFE particles (500 μg/g food) of both sizes, were dissected in 0.1 M phosphate buffer (PB, pH 7.4). Midgut samples were immediately fixed at 4 °C in a solution containing 4% paraformaldehyde and 1% glutaraldehyde in 0.15 M PB (pH 7.4). To enhance membrane contrast while minimizing non-specific background staining, post-fixation was carried out using 1% osmium tetroxide combined with 0.8% potassium hexacyanoferrate for 2 h at room temperature, followed by thorough rinsing with deionized water. Dehydration was performed through a graded acetone series. Samples were then embedded in Eponate 12™ resin (Ted Pella Inc., USA) and polymerised at 60 °C for 48 h. Ultrathin sections of the midgut were obtained using a diamond knife (Diatome 450, Switzerland), mounted on uncoated copper grids, and stained with uranyl acetate for 30 min, followed by Reynolds lead citrate for 5 min. The localisation of PTFE-MNPLs within gut compartments, including the lumen, symbiotic microbiota, and enterocytes, was examined using TEM (HITACHI H-7000, 125 kV). For haemolymph analysis, samples were directly mounted onto carbon-coated grids, air-dried, and analysed using both TEM and SEM.

(ii) Confocal Microscopy

Larvae exposed for five days to iDye-labelled PTFE-MPLs and PTFE-NPLs (500 μg/g wet food) were dissected in 1× PBS. Midgut tissues and haemolymph were stained with Hoechst 33342 (for nuclear visualisation, blue) and CellMask™ (for membrane labelling, red), both diluted 1:1000. Fluorescently labelled PTFE particles were visualised *in situ* using a Leica TCS SP5 confocal microscope (Leica Microsystems CMS GmbH, Mannheim, Germany).

### 2.4 Ex vivo degradation in larval fluid

Third-instar *Drosophila* larvae (*n* = 200; five-day-old) were collected in seven biological replicates and immediately snap-frozen in liquid nitrogen in 2 mL Eppendorf tubes. All procedures were conducted under sterile conditions to prevent contamination. For processing, tubes were kept embedded in ice, and each was supplemented with 1 mL of phosphate-buffered saline (PBS). Larvae were thoroughly homogenised using a rod homogeniser to ensure complete tissue disruption. Homogenates were centrifuged at 12,000×g for 10 min at 4 °C, and the resulting supernatants (larval fluids) were pooled into 10 mL Teflon tubes, yielding a final volume of 7 mL. PTFE-MPLs or PTFE-NPLs were then suspended in the pooled larval fluids to a final concentration of 3 mg/mL in 40 mL glass vials. Parallel control suspensions of PTFE-MPLs or PTFE-NPLs at the same concentration were prepared in PBS. All vials were tightly sealed and subjected to gentle agitation on a shaker for 72 h at room temperature. Following incubation, drops from each suspension (larval fluid or PBS) were applied to carbon-coated TEM grids. Samples were imaged using TEM to assess potential physicochemical alterations in PTFE particle morphology, as well as interactions with larval fluid components or symbiotic gut bacteria. Energy-dispersive X-ray spectroscopy (EDX) was employed to confirm the elemental composition of PTFE particles, particularly those found associated with or internalised by bacterial cells.

### 2.5 Mitochondrial membrane potential assay

Mitochondrial membrane potential (ΔΨ_m_) was assessed in larval haemocytes using the MitoProbe™ TMRM Assay Kit for Flow Cytometry (Invitrogen, Thermo Fisher Scientific). Third-instar *Drosophila* larvae (five-day-old) exposed to PTFE-MPLs or PTFE-NPLs at concentrations of 0, 125, or 500 μg/g food (triplicates) were rinsed and dissected in ice-cold PBS containing 0.07% 1-phenyl-2-thiourea (PTU; Sigma-Aldrich) to inhibit melanisation and preserve haemocyte integrity. Haemolymph from approximately 100 larvae per biological replicate was pooled and maintained on ice. For each treatment group, three biological replicates were prepared. Seven replicates were generated for the untreated controls. One unstained sample was included to assess autofluorescence, while three replicates were stained with TMRM alone (negative controls). Three additional samples were treated with 2 μM carbonyl cyanide 3-chlorophenylhydrazone (CCCP; Sigma-Aldrich) for 5 min prior to TMRM labelling to induce mitochondrial depolarization (positive controls). All samples were stained with 2 nM TMRM at 37 °C for 30 min, filtered through 45 μm cell strainers, and maintained on ice until analysis. Fluorescence was measured using a CytoFLEX flow cytometer (Beckman Coulter) with excitation/emission at 561/585 nm. A minimum of 10,000 haemocytes were analysed per sample, and data were processed using CytExpert software.

### 2.6 Transcriptomic analysis

#### 2.6.1 RNA extraction

Total RNA was extracted from *Drosophila* larvae (30 larvae per replicate, 4 biological replicates per concentration), whether treated or untreated with PTFE-MPLs or PTFE-NPLs, using the Qiagen RNeasy Mini Kit (Qiagen, Hilden, Germany) according to the manufacturer’s protocol, with modifications to accommodate downstream rRNA depletion. Although the integrity of mRNA is the primary concern for transcriptomic analyses, rRNA integrity, due to its abundance, serves as a reliable indicator of overall RNA quality. Larval tissues were homogenised in Buffer RLT containing β-mercaptoethanol to facilitate efficient cell lysis and RNase inactivation. The homogenate was clarified by centrifugation, and the resulting supernatant was combined with 70% ethanol to promote RNA binding to the silica membrane of the RNeasy spin column. Genomic DNA was removed via on-column DNase I treatment using the RNase-Free DNase Set (Qiagen). Columns were then washed with Buffer RW1 and twice with Buffer RPE to remove residual proteins and salts. RNA was eluted in RNase-free water. Prior to library preparation using the Illumina Stranded Total RNA Prep with Ribo-Zero Plus protocol, all RNA samples underwent stringent quality control. Each sample contained more than 0.5 μg of total RNA, an RNA Integrity Number (RIN) greater than 7, and a 28S/18S rRNA ratio exceeding 1, meeting Illumina’s recommended thresholds for high-quality transcriptome sequencing.

#### 2.6.2 RNA transcriptomics

Total RNA samples were quantified and sent to Macrogen Inc. (Seoul, South Korea) for high-throughput sequencing on the Illumina platform (paired-end, 2 × 150 bp). Each sample generated approximately 40 million reads on average, providing robust data for downstream bioinformatic analyses.

#### 2.6.3 Data processing and analysis

Data analyses were performed using R (v4.3.3) and RStudio (v2023.06.1) software. Raw reads were quality-filtered with Rfastp package; adapters and low-quality bases were removed with the default parameters. Reads shorter than 20 bp were filtered. Filtered reads were aligned to the *D. melanogaster* genome (release 6 from FlyBase) using Rsubread package with default parameters. Gene expression was quantified using Rsubread package, low-expression features were filtered with the edgeR function filterByExpr, and counts were normalized using the edgeR function calcNormFactors. Batch effects were corrected using surrogate variable analysis (SVA) implemented in the sva package. Differentially expressed genes (DEGs) for each treatment and concentration were identified with the voom/limma packages. Enriched functional terms were determined through over representation analysis (ORA) and gene set enrichment analysis (GSEA) using clusterProfiler package. Several data collections were used as models: Gene Ontology (GO), Kyoto Encyclopedia of Genes and Genomes (KEGG), Reactome, WikiPathways and MSigDB Hallmark collection. GO functional terms were divided by ontology. Biological Process results were reduced to representative terms through the simplify function with a cutoff value of 0.5. Overlapping Cellular Component terms were filtered out to ensure clearer visualisation. Data visualisation employed the ggplot2 package. A diagram representing the described pipeline is included in Fig. S10.

#### 2.6.4 Quantitative real-time PCR

To validate representative differentially expressed genes from the transcriptomic analysis, quantitative real-time PCR (qRT-PCR) was performed. Total RNA of larval samples (30 larvae per sample, four replicates) was reverse transcribed into complementary DNA (cDNA) using the PrimeScript RT kit with gDNA Eraser (Vazyme Biotechnology Co., Ltd., Nanjing, China). The resulting cDNA was diluted 10-fold and used as a template for qRT-PCR with AceQ Universal SYBR qPCR Master Mix (Vazyme) on a CFX Connect Real-Time PCR Detection System (Bio-Rad, Singapore). Each reaction was performed in duplicate to minimize technical variability. Relative gene expression levels were calculated using the 2^−*ΔΔCt*^ method, with Actin (Act) serving as the internal reference gene. The list of primers used is indicated in Table S2.

### 2.7 Oxidative stress induction

To assess oxidative stress induced by PTFE-MNPLs, reactive oxygen species (ROS) levels were measured in *Drosophila* haemocytes. Haemolymph was collected from 5-day-old larvae (about 50 pooled from triplicates per concentration) and incubated with 5 μM DCFH-DA. Fluorescence was detected using a microscope with 485 nm excitation and 530 nm emission filters. Image analysis was performed in ImageJ, applying thresholds to exclude background fluorescence and ensure that only signals from DCF-stained haemocytes were quantified. ROS levels were calculated as the mean fluorescence intensity of 10 randomly selected fields per concentration and compared to unexposed controls. Haemocytes treated with 0.5 mM H_2_O_2_ for 30 min served as a positive control. ROS intensities were compared to autofluorescence controls to confirm that observed increases reflected genuine ROS production rather than background or imaging artifacts.

### 2.8 Genotoxic damage induction

The genotoxic potential of PTFE-MNPLs was evaluated in *Drosophila* haemocytes using the comet assay, which measures the percentage of DNA migrating from the nucleus to the tail. Third-instar larvae (72-h-old) were exposed for 24 h to PTFE-NPLs or PTFE-MPLs incorporated into instant medium at concentrations of 125 and 500 μg/g wet food. Milli-Q water and 4 mM EMS were used as negative and positive controls, respectively. Haemolymph was extracted from approximately 100 larvae per concentration, pooled from three biological replicates, and mixed with 0.75% low-melting-point agarose (1:9) at 37 °C. Drops of the haemolymph-agarose mixture were applied to Gel Bond film (GBF) and lysed for 1 h at 4 °C in the dark. DNA unwinding was carried out in a cold alkaline electrophoresis buffer (0.001 M EDTA, 0.3 M NaOH, pH 13.2) for 25 min, followed by electrophoresis for 20 min at 20 V and 300 mA. GBFs were then neutralised with two washes in 1% PBS (5 min each) and a 1-min wash in distilled water. DNA was dehydrated in ethanol, stained with SYBR Gold, and analysed using the Komet 5.5 image analysis system (Kinetic Imaging Ltd., Liverpool, UK). From each replicate, 100 individual comets were scored, and consistency was maintained across replicates.

### 2.9 Statistical analysis

Data were analysed using GraphPad Prism 9 software (GraphPad Software Inc., CA, USA). The assumption of normality was assessed using the Shapiro-Wilk test. The paired *t*-tests, one-way ANOVA, or two-way ANOVA (parametric tests) with Tukey’s multiple comparison test was applied for normally distributed data. Non-normally distributed data were analysed using the Kruskal–Wallis test with Dunn’s multiple comparisons or the Wilcoxon/Mann–Whitney *U* tests, as appropriate. Normally distributed data were represented as mean ± standard deviation, while the median and interquartile range (Box and violin) represented the nonparametric data. Statistical significance was defined as ∗*p* ≤ 0.05, ∗∗*p* ≤ 0.01, ∗∗∗*p* ≤ 0.001, and ∗∗∗∗*p* ≤ 0.0001. Sample size and replicate numbers varied depending on the experimental technique; however, all assays were performed with at least three independent biological replicates (*n* refers to the number of individuals). RT-PCR analyses were additionally conducted with two technical replicates per sample to ensure measurement reliability.

## 3 Results

### 3.1 PTFE-MNPLs characterization

To investigate how particle properties govern biological behaviour, we first performed comprehensive physicochemical characterisation of PTFE-MNPLs. SEM and TEM revealed that PTFE-MPLs (2186.73 ± 1372.11 nm) formed irregular, polydisperse plate-like structures, whereas PTFE-NPLs (249.41 ± 60.12 nm) were uniform, spherical, and markedly smaller (Fig. S1a–f). Surface topography differed: MPLs were fractured and angular, while NPLs displayed smooth, well-defined boundaries, likely affecting dispersibility and cellular interactions. In aqueous suspension, MPLs rapidly aggregated into large clusters (9113.33 ± 2023.01 nm), whereas NPLs remained colloidally stable, with hydrodynamic diameters below 500 nm (486.57 ± 20.29 nm) (Fig. S1g). Dynamic light scattering (DLS) measurements were reported as Z_a_ᵥ_e_ values. For MPLs, the number-based mean (approximately 101 nm) indicated a minor fraction of fine particles, while the majority remain as large aggregates, consistent with Z_a_ᵥ_e_ and SEM data. In contrast, NPLs exhibited a number-based mean of approximately 211 nm, confirming that most particles are within the nanoscale range. Zeta potential measurements indicated weak surface charge for MPLs (−2.42 ± 0.11 mV) compared with NPLs (−39.2 ± 1.16 mV), consistent with enhanced electrostatic stabilisation and reduced aggregation of nanoparticles (Fig. S1h). FTIR spectroscopy confirmed the chemical integrity of both particle types via characteristic C–F and C–C vibrational bands (Fig. S1i and j), and EDX analysis detected strong fluorine signals, validating their fluoropolymer composition (Fig. S1k,l). These contrasting structural and surface features suggest that MPLs and NPLs may differ profoundly in environmental persistence, bioavailability, and cellular trafficking.

To establish a physiologically relevant exposure paradigm, we next evaluated particle toxicity throughout the life cycle of *D. melanogaster*. Eggs were deposited on standard media supplemented with PTFE-MPLs or PTFE-NPLs at graded concentrations (31.25, 125, and 500 μg/g wet food) (Fig. S2a). Developmental viability, assessed as the proportion of eggs reaching adulthood, was unaffected across all exposure levels relative to controls (Fig. S2b). Adults exhibited no overt morphological abnormalities at any concentration, supporting the selection of the two highest concentrations (125 and 500 μg/g wet food) for subsequent analyses. These results define a non-lethal yet biologically relevant exposure window, suitable for probing particle interactions at cellular and molecular resolution.

### 3.2 Polytetrafluoroethylene particles traverse the gut, interact with bacteria, partially degrade, and compromise tissue integrity

#### 3.2.1 Inside midgut lumen

The biodistribution, dynamics, and interactions of orally administered PTFE particles (MPLs and NPLs) in *D. melanogaster* larvae were tracked using high-resolution electron and confocal microscopies (Fig. 1, Fig. 2, and S3–S8). TEM imaging (Fig. 1a–t) revealed the progressive trajectory of both particle types, from their dispersion within the gut lumen to translocation across the intestinal barrier and eventual detection in the haemolymph. These findings were further corroborated by confocal microscopy (Fig. 1u–y). Initially, both PTFE particle types localised to the gut lumen, where they exhibited extensive interactions with the resident gut microbiota and became enclosed by intestinal vacuoles or associated with the peritrophic membrane (Fig. 1a–e for MPLs; Fig. 1k–o for NPLs). PTFE-MPLs appeared irregularly dispersed, whereas NPLs appeared as distinct black dots. Detailed ultrastructural analyses revealed PTFE particle interactions with midgut symbiotic bacteria, shown for MPLs (Fig. S3a–d) and for NPLs (Fig. S3e–h). According to Fig. S3, PTFE particles were observed near or attached to bacterial surfaces (a,b,e,f), followed by progressive uptake and internalisation (c,g), mediated by pronounced morphological alterations, including membrane disruption and structural damage (d,h) for MPLs and NPLs, respectively. These findings suggest that PTFE particles may pose a hazard to beneficial gut microbiota by disrupting their habitat, highlighting potential implications for intestinal health.

**Fig. 1:**
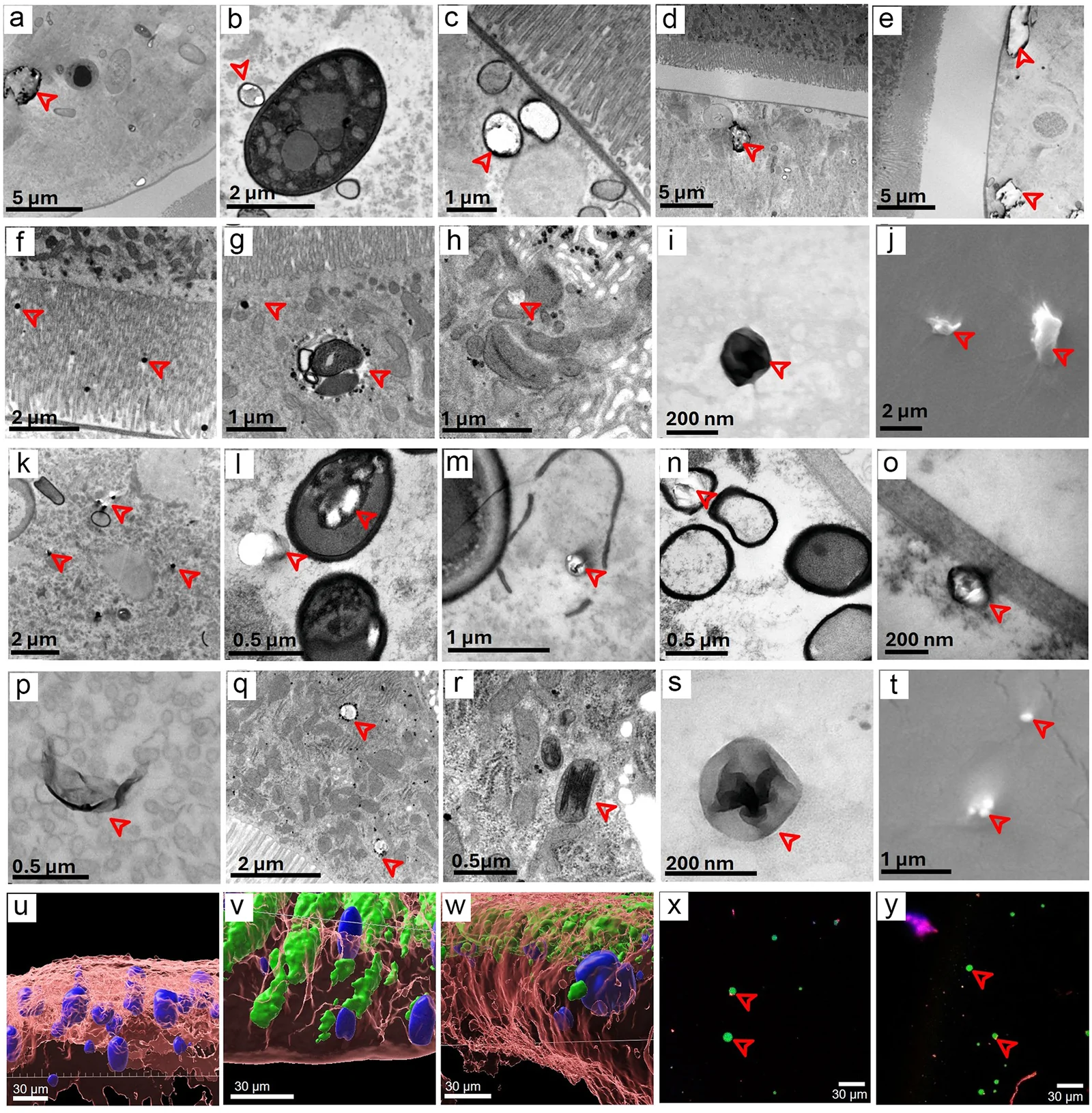
Biodistribution and cellular effects of orally administered PTFE particles in *Drosophila* larvae. (a–j) TEM imaging of PTFE-MPL internalisation pathway. Particles are dispersed throughout the midgut lumen (a), attached to resident microbiota (b), enclosed within vesicle-like structures (c), and interact with the peritrophic matrix (d, e). During epithelial translocation, they interact with microvillar membranes (f) and accumulate intracellularly (g). These interactions cause ultrastructural damage, including mitochondrial swelling and disruption (h). PTFE-MPLs breach the intestinal–haemolymph barrier and enter systemic circulation, as shown by TEM (i) and SEM (j). (k–t) PTFE-NPL biodistribution: in the midgut lumen (k), associated with or internalised by microbiota (l), partially or fully enclosed in vesicular compartments (m, n), and interacting with the peritrophic matrix (o). Internalised NPLs cross enterocytes that induce histopathological alterations and disrupt microvilli (p), that undergo lysosome-like digestion (q), and that undergo mitochondrial damage with condensed or fragmented cisternae (r). NPLs ultimately cross the epithelial barrier and reach the haemolymph (s, t). (u–y) Confocal fluorescence imaging. Control larvae show no signal (u), whereas MPL- and NPL-treated larvae exhibit widespread fluorescence in the intestinal epithelium, including perinuclear regions (v, w), and in the haemolymph (x, y), respectively, indicating systemic distribution. Red arrows indicate PTFE-MNPLs.

**Fig. 2:**
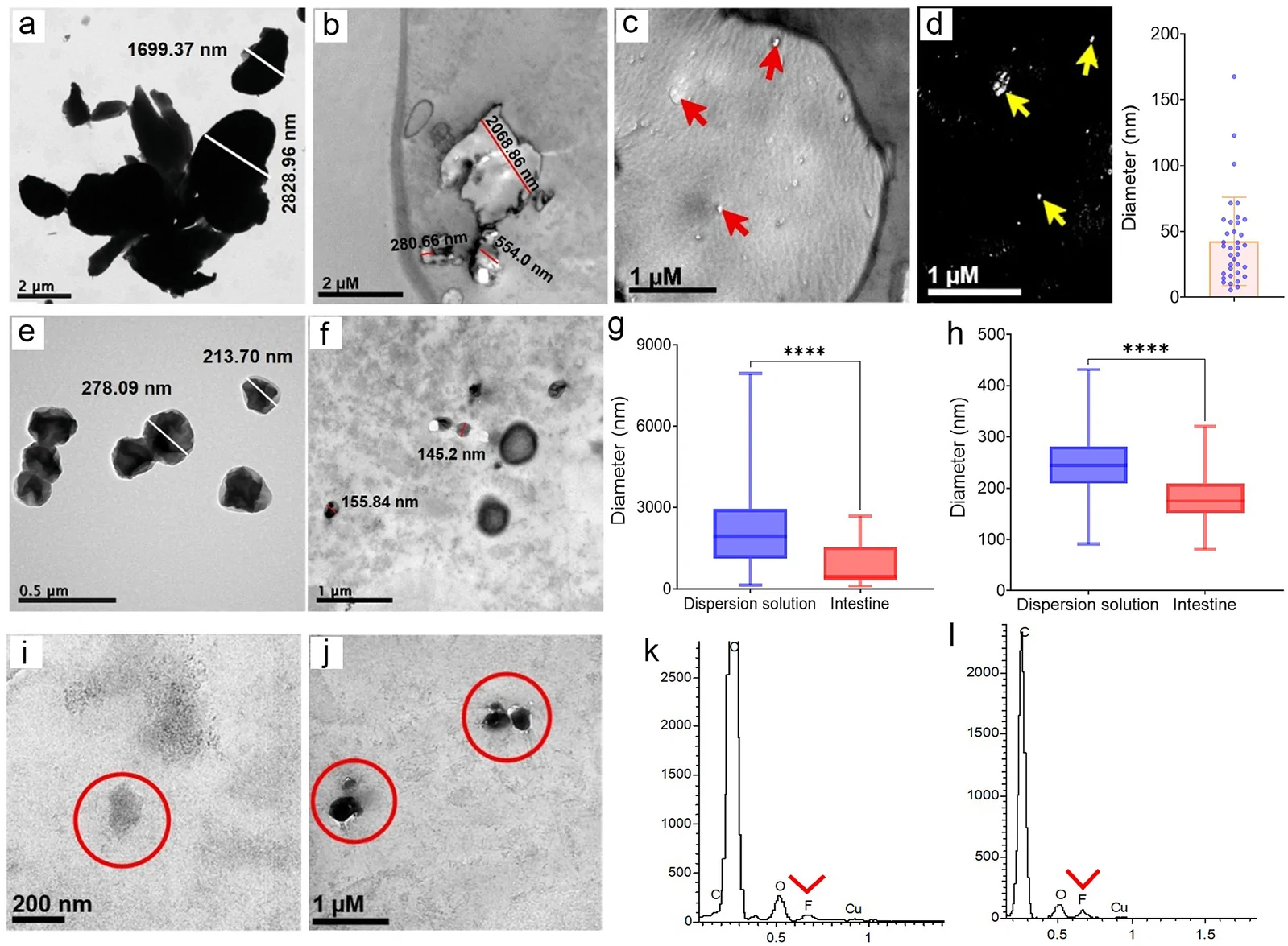
Bio-erosion of PTFE-MPLs and PTFE-NPLs following oral administration in *Drosophila* larvae. (a) TEM image of a PTFE-MPLs dispersion solution, illustrating the initial microparticle morphology. (b) PTFE-MPLs localised within the midgut lumen near the peritrophic membrane, exhibiting surface degradation and diameter reduction. (c) Enlarged TEM view of PTFE-MPLs in the larval gut lumen revealing surface erosion and the presence of nanopores, suggestive of nanoscale oligomer leaching. (d) Threshold-adjusted TEM imaging highlights leached nanosized oligomers (appearing as white dots) originating from PTFE-MPLs; the average diameter of these oligomers is 46.67 ± 34.96 nm. (e) TEM image of the PTFE-NPLs dispersion solution, showing their initial nanostructure. (f) PTFE-NPLs identified in proximity to the peritrophic membrane within the midgut lumen, also exhibiting surface degradation. Boxplots of particle size distributions for PTFE-MPLs (g) and PTFE-NPLs (h) before and after internalisation reveal a pronounced decrease in particle diameter, consistent with biological erosion of PTFE. TEM–EDX elemental analysis of internalised PTFE-MPLs (i) and PTFE-NPLs (j) in larval intestinal tissue confirms the presence of fluorine, consistent with the characteristic chemical composition of PTFE. Fluorine signal further validates the internalisation of both MPLs (k) and NPLs (l) PTFE particles. Statistical analysis was performed using the Mann–Whitney test to compare PTFE-MPLs and PTFE-NPLs in both intestinal samples and dispersion solutions (∗∗∗∗*p* ≤ 0.0001).

In parallel, interactions with other intestinal effectors further contributed to particle modification. As a result, particles exhibited reduced diameter and increased fragility, with superficial pores forming on their surfaces (Fig. 2), particularly evident in microsized particles (Fig. S4). Quantitative analysis revealed a significant reduction in particle size following internalisation into the midgut lumen, decreasing from a median of 1944.1 [interquartile range (IQR): 1827.6] to 459.4 (IQR: 1225.3) nm for PTFE-MPLs and from 243.5 (IQR: 74.0) to 174.4 (IQR: 57.5) nm for PTFE-NPLs, indicating biological erosion of the PTFE particles (Fig. 2a–h). Fragmentation of MPLs further generated numerous detached particles with an average size of 46.67 ± 34.96 nm. The PTFE identity of these particles was confirmed by EDX analysis (Fig. 2i–l). Fig. S4 illustrates that all MPLs distributed within the midgut lumen of exposed larvae exhibited fragility (c–e). Numerous pores were visible, indicating detachment of smaller fragments (f–h), in contrast to the well-compacted particles present before larval exposure (a). The lumen of unexposed larvae appeared devoid of any PTFE particles (b).

To confirm the effects of larval fluid interactions on PTFE particles, *ex vivo* experiments were conducted for MPLs (Fig. S5) and NPLs (Fig. S6). Fig. S5 demonstrates that PTFE-MPLs appeared well-structured and morphologically intact in PBS solution (a). In contrast, incubation with larval fluids induced progressive erosion, pore formation, and fragmentation (b–g). TEM–EDX analysis confirmed the presence of fluorine in PTFE-MPLs within larval fluids (h) and inside bacteria (i), consistent with PTFE. Particle size was markedly reduced, with median (IQR) values decreasing from 2346.5 (2131.0) to 572.7 (2181.6) nm (j), accompanied by the formation of surface pores averaging 251.52 ± 84.83 nm in diameter (k). A similar finding was observed in the case of PTFE-NPLs (Fig. S6a–j). Particle size was also substantially decreased from 249.85 ± 61.93 nm to 214.1 ± 53.98 nm, with surface pores averaging 45.96 ± 13.97 nm. The larval fluids contained a mixture of host-derived enzymes, proteins, and symbiotic gut microbiota, which may collectively contribute to the observed PTFE particle transformations.

#### 3.2.2 Crossing the intestinal barrier

After localisation in the midgut lumen, PTFE particles crossed the intestinal barrier, initiating a new phase of interactions and biological responses (Figs. 1, S7). Translocation of PTFE-MNPLs occurred primarily via intestinal vacuoles or by direct passage across the chitinous peritrophic membrane. MPLs were fully encapsulated within intestinal vacuoles, bringing them into direct contact with the peritrophic membrane (Figs. 1c and S7a,b), and subsequently passed into the cytoplasm of midgut cells. Smaller fragments, visible as black dots, were frequently observed within the microvilli and cytoplasm of enterocytes (Figs. 1f,S7c). In the case of NPLs, they were initially surrounded by intestinal membranes before being enclosed within compact vacuoles (Figs. 1f and S7e–g). Notably, an alternative internalisation pathway was also identified, particularly for nanosized plastic particles, involving their direct penetration through the intestinal barrier (Fig. 1o).

#### 3.2.3 Intracellular and systemic distribution patterns

In enterocytes, PTFE-MNPLs displayed widespread distribution and extensive interaction with these primary digestive cells of the larval midgut (Figs. 1, S8). MPLs exhibited a broad intracellular distribution, appearing either as large particles localised within lysosome-like compartments or as dense black dots, likely representing small fragments detached from particle surfaces (Fig. S8a and b). Internalisation of NPLs led to evident damage to the microvilli and plasma membrane of enterocytes, with the particles appearing as black dots or enclosed within lysosome-like vesicles (Figs. 1p,q and S8e,f). MPLs were observed either surrounded by or localised within mitochondria (Figs. 1g,h and S8c,d), and similar localisation was noted for NPLs (Figs. 1r and S8g,h). These interactions caused pronounced ultrastructural alterations, including mitochondrial swelling, cristae disruption, vacuolation, and abnormal morphological features. Ultimately, PTFE particles accumulated within the haemolymph, where they were dispersed and established direct contact with haemocytes. The presence of PTFE-MNPLs was confirmed through TEM and SEM analyses (Fig. 1i and j for MPLs and Fig. 1s,t for NPLs, respectively). Confocal fluorescence imaging (Fig. 1u–y) further supported the ultrastructural findings obtained from TEM analyses. No detectable fluorescence signal was observed in control larvae (u). In contrast, larvae exposed to MPLs and NPLs exhibited strong and widespread fluorescence throughout the intestinal epithelium, particularly in the perinuclear regions (v,w). Moreover, fluorescence was also detected within the haemolymph (x,y), confirming the systemic distribution of PTFE particles beyond the intestinal barrier.

### 3.3 Size- and concentration-dependent transcriptomic effects of PTFE micro- and nanoplastics in Drosophila

Transcriptomic profiling of *Drosophila* larvae exposed to PTFE-MPLs and PTFE-NPLs at 125 and 500 μg/g wet food revealed concentration- and size-dependent transcriptional responses. A Venn diagram showed differentially expressed genes (DEGs) unique to or shared between PTFE-MPL- and PTFE-NPL-exposed samples (Fig. S9a). At 125 μg/g wet food, MPL exposure induced 158 DEGs compared with 975 DEGs for NPLs, whereas at 500 μg/g wet food, MPLs caused 553 DEGs versus 2897 DEGs for NPLs, indicating a concentration- and particle size-dependent effect (Fig. S9b). Gene Set Enrichment Analysis (GSEA) identified deregulated pathways following PTFE exposure, with distinct enrichment patterns for MPLs and NPLs at both concentrations according to various databases (Fig. S9c). Over Representation Analysis (ORA) highlighted key functional categories and biological processes enriched in the DEGs affected by PTFE (Fig. S9d). GSEA of GO Cellular Component terms revealed deregulation of cytoskeletal, plasma membrane, cell junction, mitochondrial, lysosomal, and endosomal components by both PTFE particles (Fig. S9e). ORA of GO Biological Process terms and GSEA of KEGG pathways illustrated pathway enrichment and deregulation, respectively, across treatments, showing PTFE particles exposure affected gene expression in several processes, mainly related to metabolism, particularly for NPLs (Fig. S9f and g).

Volcano and scatter plots were generated to visualize the distribution of DEGs in terms of up- and down-regulation following exposure to PTFE-MPLs and PTFE-NPLs (Fig. 3a and b). Both plots revealed distinct sets of up- and down-regulated transcripts, with a pronounced concentration- and size-dependent enrichment of highly perturbed genes upon exposure to PTFE particles, particularly evident for NPLs (Fig. 3a). A comparable trend was observed in the scatter plot (Fig. 3b). Notably, the number of downregulated genes exceeded that of upregulated genes across all conditions. Specifically, downregulated genes numbered 134, 482, 793, and 1838, while upregulated genes numbered 24, 71, 182, and 1059 for MPLs and NPLs at 125 and 500 μg/g wet food, respectively. Of a total of 3021 DEGs identified following exposure to PTFE-MNPLs, 110 genes were consistently deregulated across all concentrations and particle sizes. Their expression levels compared to the untreated samples were depicted using a heat map (Fig. S10). Analysis revealed uniform expression patterns across all treatments, with effects generally increasing at higher concentrations. This gene set included transcripts with well-characterised biological functions (Fig. S10a) as well as genes with uncharacterised or unknown functions (Fig. S10b).

**Fig. 3:**
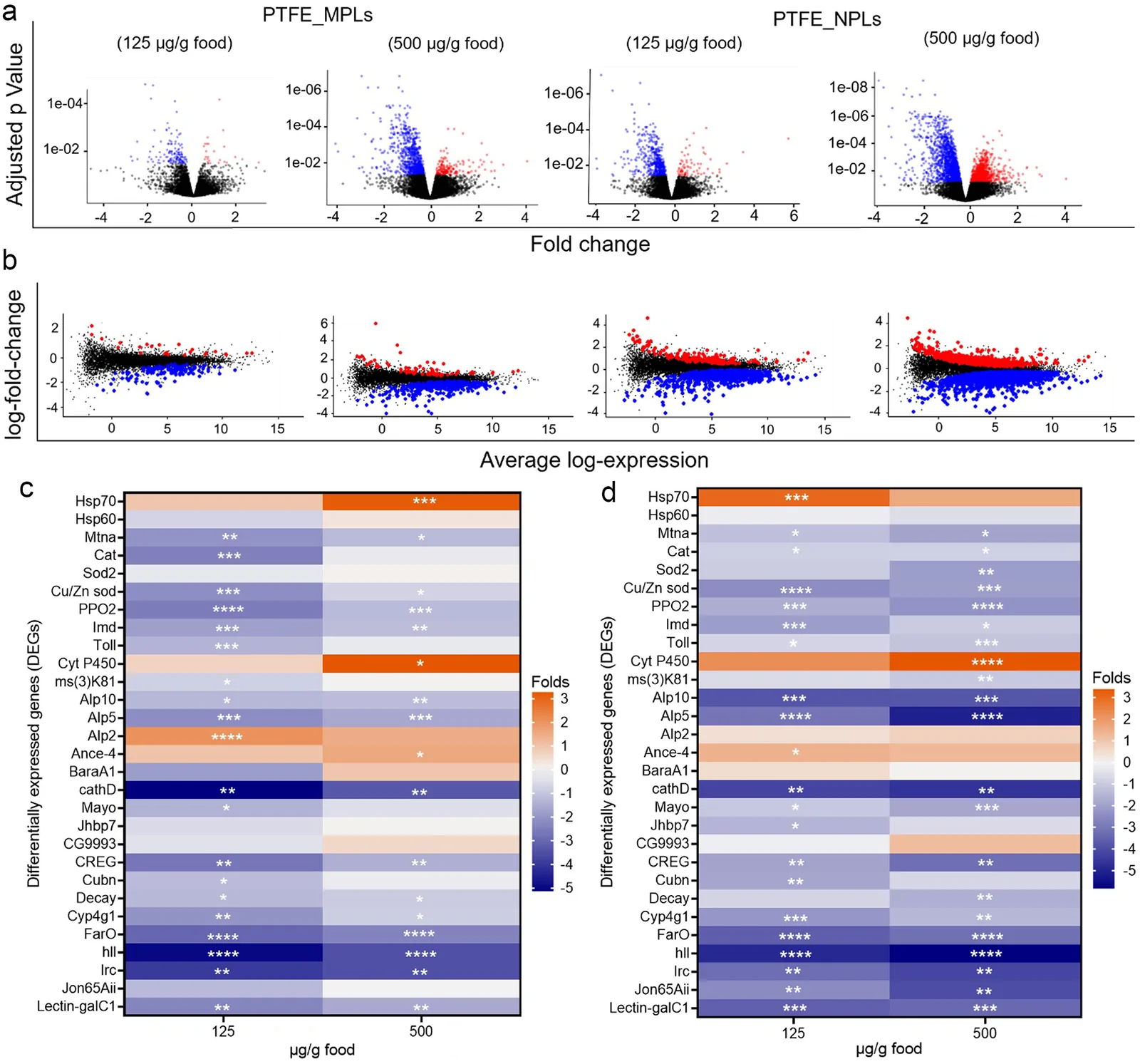
Transcriptomic alterations following PTFE-MNPL exposure. (a) Volcano plots of genome-wide differential gene expression in larvae exposed to PTFE-MPLs or PTFE-NPLs at 125 and 500 μg/g wet food. The x-axis shows log_2_ fold change; the y-axis shows –log_10_ adjusted *p*-value. (b) Scatter plots of mean expression versus log_2_ fold change represent mean expression across biological replicates. Red and blue dots indicate significantly up- and downregulated genes, respectively. PTFE-NPLs caused more extensive transcriptomic changes than PTFE-MPLs. RT–qPCR validation of selected genes for PTFE-MPLs (c) and PTFE-NPLs (d), confirming transcriptomic results. Statistical significance was determined using two-way ANOVA with Tukey’s multiple comparisons test (*∗p* ≤ *0.05, ∗∗p* ≤ *0.01, ∗∗∗p* ≤ *0.001, ∗∗∗∗p* ≤ *0.0001*).

Transcriptomic profiling of the 110 common DEGs revealed that PTFE-MNPL exposure affected multiple interconnected biological pathways, suggesting broad physiological stress. Genes involved in metabolism and mitochondrial function (*Odc1, hll, HR38, FarO, Cyp4g1, phu*), together with lysosomal and autophagy regulators (*vhaM9.7-b, cathD, Npc1b, Gba1b, Cubn, Rab32*), were markedly deregulated, suggesting impaired energy balance and compromised intracellular degradation. A suite of inflammation- and metabolism-associated genes (*Alp2, Alp5, Alp8, Alp9, Alp10*), along with antioxidant (*Gclm, Fer1HCH*) and immune response genes (*LysC, PGRP-SC1a, Upd2, NimB5, VIR-1, IRC, Ag5r2, PPO2*), exhibited altered expression patterns indicative of oxidative stress and disorder in immune activity. PTFE exposure further affected endocrine (*Ance-4, Jhbp7*), neuromuscular and neuronal (*Bace, sls, Ugalt, hlk, nrm, Ddr, Lectin-galC1*), and epigenetic regulators (*Gclm, Nurf-38, lncRNAs, asRNAs, Sage*), accompanied by modulation of apoptosis- and tissue integrity-related genes (*Decay, Mipp1, Tsap29fb, PAICS, Jon9*9Ci*, Jon65Aii*). Collectively, these transcriptomic alterations reveal that PTFE-MNPL exposure triggers extensive, multi-system dysregulation spanning metabolic, immune, endocrine, and neuronal processes.

RT-PCR analysis (Fig. 3c and d) was performed to validate the pathways represented by the common DEGs and to further evaluate the broader set of DEGs (2897) identified under high-concentration PTFE-NPL exposure. Most validated genes exhibited downregulation, particularly pronounced in response to NPL treatment. Genes related to metabolism and mitochondrial activity (*hll, FarO, Cyp4g1*) and those associated with lysosomal and autophagy processes (*cathD*) were significantly affected, confirming disrupted energy homeostasis and impaired intracellular degradation consistent with the transcriptomic results. Inflammation- and metabolism-associated genes (*Alp2, Alp5, Alp10*), along with antioxidant regulators (*Cu/Zn SOD, Sod2, Cat, Mtna*), displayed altered expression patterns indicative of oxidative stress and immune activation. Moreover, PTFE-MNPL exposure led to the deregulation of immune mediators (*IRC, PPO2, Toll, Imd, BaraA1*), endocrine components (*Ance-4, Jhbp7*), neuromuscular genes (*Lectin-galC1*), and reproductive genes [*ms(3)K81*], as well as genes involved in proliferation, differentiation, and apoptosis (*Decay, CREG, Jon65Aii*). Stress-responsive genes (*Hsp70Aa, Hsp60A, Cyt P450, Mayo, CG9993*) were also markedly affected, collectively confirming that PTFE-MNPL exposure, particularly NPLs exposure, induces broad, multi-system perturbations spanning metabolic, immune, endocrine, and cellular stress pathways. Thus, RT-PCR analysis strongly supports the transcriptomic findings.

### 3.4 Mitochondrial structure and function following PTFE-MNPL exposure

Building on the transcriptomic evidence of organelle stress, we next investigated the effects of PTFE particle internalisation on mitochondrial integrity, combining ultrastructural imaging, gene expression profiling, and functional assays. TEM revealed pronounced mitochondrial damage in PTFE-exposed larvae, characterised by swelling, condensed or fragmented cristae, and ruptured outer membranes (Fig. 4a and b). Notably, some mitochondria displayed a distorted, kidney-shaped morphology in close association with internalised PTFE-MPLs (Fig. 4b). These structural lesions mirror earlier observations of PTFE particles adjacent to or within mitochondria (Figs. 1h,r and S8). Consistent with these ultrastructural findings, GSEA of whole-larval transcriptomes revealed significant downregulation of oxidative phosphorylation and mitochondrial metabolic pathways, with the effect being particularly pronounced following PTFE-NPL exposure (Figs. 4c,S9). Specifically, a broad set of genes encoding subunits of mitochondrial respiratory chain complexes I, II, III, and IV were transcriptionally altered following PTFE-NPL exposure, indicating disrupted electron transport and reduced ATP production (Fig. 4d).

**Fig. 4:**
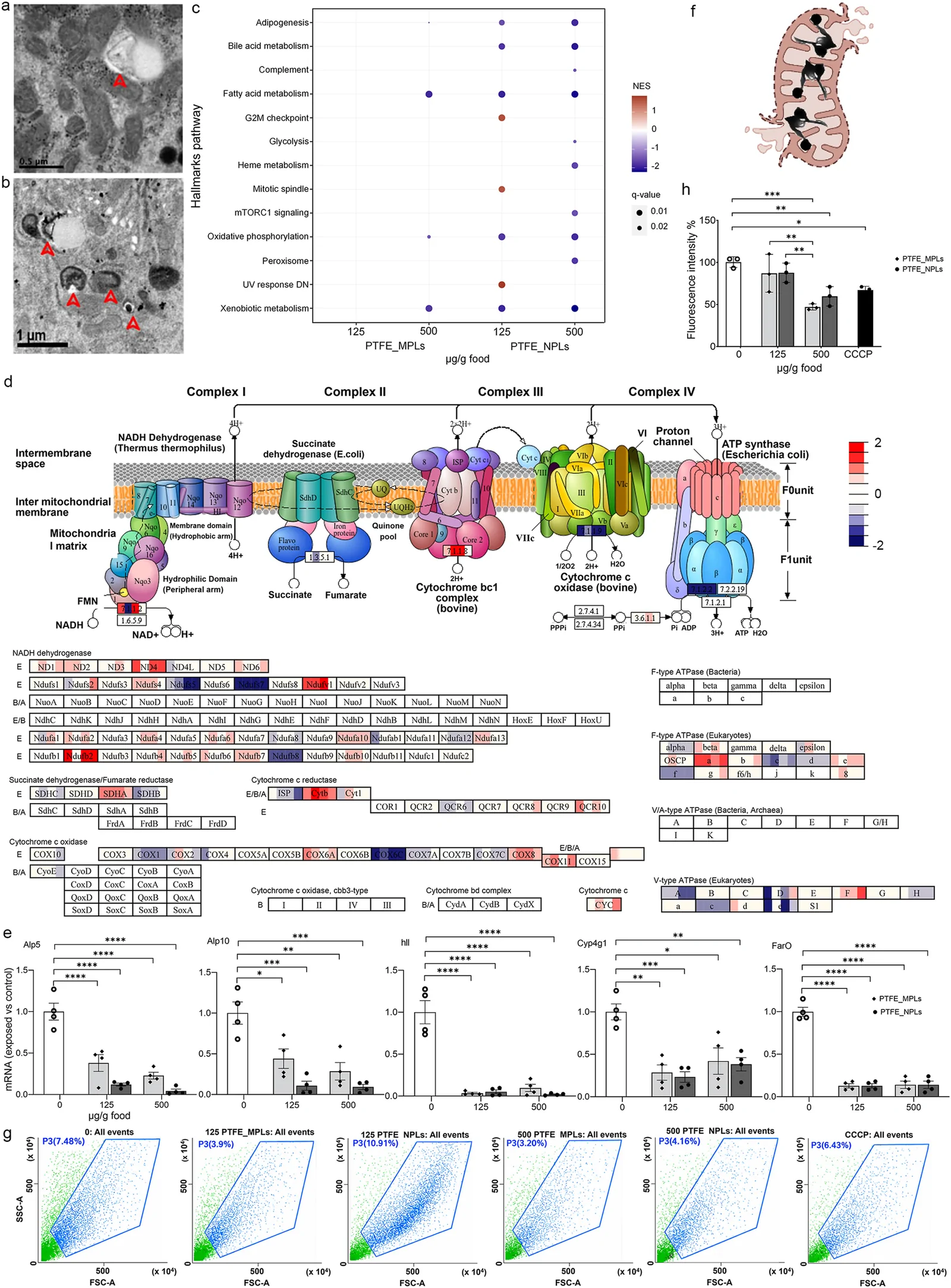
Mitochondrial structure and function following PTFE-MNPL exposure. (a, b) TEM images show mitochondrial damage associated with PTFE-MPLs and PTFE-NPLs internalisation, respectively. (c) GSEA shows marked downregulation of oxidative phosphorylation and related metabolic pathways, especially with NPLs. (d) KEGG oxidative phosphorylation pathway of *D. melanogaster*, showing fold-change values for PTFE-MNPLs treatments (125 and 500 μg/g) in each square. Transcriptomic profiling demonstrates broad suppression of genes encoding subunits of mitochondrial respiratory chain complexes I, III, IV, and V, indicating impaired electron transport and ATP production. (e) PTFE exposure elevates mitochondrial stress-responsive genes, including *Alp5*, *Alp10*, *FarO*, *Cyp4g1*, and *hll*. (f) A schematic illustrates PTFE-induced disruption of mitochondrial membrane integrity. (g) Flow cytometry of TMRM-stained haemocytes shows a concentration-dependent decrease in mitochondrial membrane potential, quantified as mean fluorescence intensity (MFI) in panel (h), indicating concentration-dependent mitochondrial membrane disruption following PTFE uptake. Data are presented as mean ± SD. Statistical significance was assessed by two-way ANOVA with Tukey’s multiple comparisons test (*∗p* ≤ 0.05, *∗∗p* ≤ 0.01, *∗∗∗p* ≤ 0.001, *∗∗∗∗p* ≤ 0.0001). CCCP, carbonyl cyanide 3-chlorophenylhydrazone; FSC-A, Forward Scatter-Area; SSC-A, Side Scatter-Area.

Expression of key metabolism- and mitochondrial-related genes (*hll, FarO, Cyp4g1*), along with metabolism- and inflammation-associated genes (*Alp5*, *Alp10*), was significantly downregulated, reflecting the mitochondrial damage associated with PTFE-MNPLs (Fig. 4e). This damage may lead to disruption of mitochondrial membrane potential (Fig. 4f). To functionally validate mitochondrial impairment, we assessed mitochondrial membrane potential in larval haemocytes using tetramethyl rhodamine methyl ester (TMRM) staining and flow cytometry. A clear concentration-dependent reduction in TMRM fluorescence was observed upon PTFE-MNPL exposure (Fig. 4g and h). These results suggest a loss of mitochondrial polarization, a hallmark of mitochondrial dysfunction, associated with the presence of internalised PTFE particles.

### 3.5 PTFE-MNPLs elicit oxidative stress and genotoxicity in Drosophila larvae haemocytes

Exposure of *Drosophila* larvae to PTFE-MPLs and PTFE-NPLs triggered oxidative stress in circulating haemocytes (Fig. 5). Transcriptomic and pathway enrichment analyses revealed widespread dysregulation under PTFE-NPL exposure, particularly affecting the proton-transporting V-type ATPase complex and mitochondrial complexes I and II (Fig. 5a). Perturbation of these key mitochondrial functions may contribute to the observed oxidative stress, as illustrated in the schematic (Fig. 5b). RT–qPCR revealed significant downregulation of key antioxidant genes, including *Cu/Zn Sod, Sod2, Cat,* and *Mtna*, irrespective of concentration or particle size (Fig. 5c), reflecting impaired detoxification pathways.

**Fig. 5:**
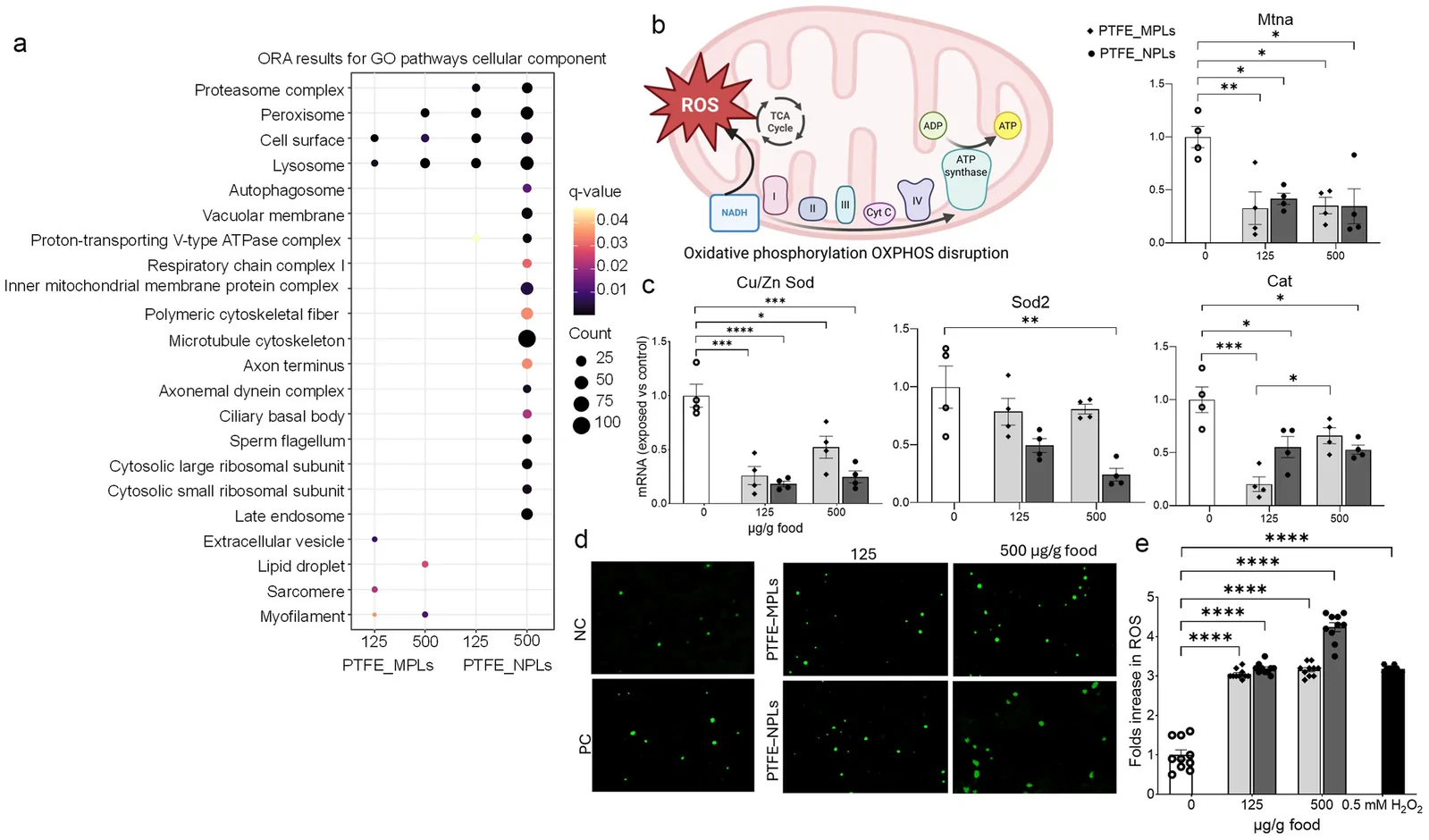
PTFE-MNPLs induce oxidative stress in *Drosophila* larval haemocytes. (a) ORA bubble plot showing enriched cellular component GO terms, with widespread dysregulation under PTFE-NPL exposure, particularly in ATPase and mitochondrial complexes I and II. Bubble size indicates gene count; colour denotes *q*-value significance. (b) Schematic illustration depicting how mitochondrial damage and TCA cycle activity contribute to ROS generation. (c) RT–qPCR analysis of oxidative stress-related genes revealed significant downregulation of *Cu/Zn Sod, Mtna, Catalase,* and *Sod2*, irrespective of concentration or particle size. (d) Representative fluorescence microscopy images of DCF-DA–stained haemocytes showing elevated ROS production in PTFE-exposed larvae, with increased fluorescence intensity corresponding to higher oxidative stress levels. (e) Quantification of fluorescence intensity demonstrates a concentration-dependent increase in ROS, most pronounced at high PTFE-NPL exposure. Data are presented as mean ± SD; statistical significance was determined using two-way ANOVA with Tukey’s multiple comparisons test (∗*p* ≤ 0.05, ∗∗*p* ≤ 0.01, ∗∗∗*p* ≤ 0.001, ∗∗∗∗*p* ≤ 0.0001. ORA, Over-Representation Analysis; GO, Gene Ontology; TCA, Tricarboxylic Acid; DCF-DA, 2′,7′-Dichlorofluorescin Diacetate; NC, Negative Control; PC, Positive Control; ROS, Reactive Oxygen Species.

Consistently, DCF-DA–stained haemocytes from PTFE-MNPLs–exposed larvae exhibited elevated ROS levels, increasing in a concentration-dependent manner (Fig. 5d). Quantitative analysis confirmed that ROS production occurred regardless of particle size; however, PTFE-NPLs induced over 4-fold higher ROS compared with untreated larvae and approximately 1-fold higher than PTFE-MPLs at comparable concentrations (Fig. 5e). Together, these findings indicate that both micro- and nanoscale PTFE particles disrupt haemocytes redox homeostasis, with nanoparticles exerting a more pronounced pro-oxidant effect.

To evaluate the genotoxic potential of PTFE-MNPLs in *Drosophila* larvae, haemocytes were examined as primary cellular targets, given that PTFE particles can reach the haemolymph and associate with these cells (Fig. 6). Fig. 6a–c illustrates the interaction of PTFE-MNPLs with exposed larvae and their ability to localise near the nucleus. TEM analysis further confirmed the internalisation of PTFE particles in proximity to the nuclei of midgut cells (Fig. 6c). For the comet assay, unlike enterocytes, haemocytes were chosen as single, non-tissue cells to allow direct assessment of DNA damage at the individual cell level and to avoid additional damage that may occur in isolated tissue cells. However, the presence of PTFE-MNPLs near the nucleus may potentially contribute to DNA damage (Fig. 6d). The degree of genotoxicity increased proportionally with the percentage of DNA fragmentation, as observed in representative comet assays of haemocytes exhibiting mild, moderate, and severe DNA damage (Fig. 6e). Exposure to PTFE-MNPLs induced DNA damage in a concentration- and size-dependent manner, with a more persistent effect observed for NPLs (Fig. 6f). These molecular events culminated in broader cellular dysfunctions, with PTFE exposure disrupting key regulators of apoptosis, cell migration, and tissue integrity, as evidenced by transcriptomic changes in genes such as *Decay*, *Mipp1*, *Tsap29fb*, *CREG*, *Paics*, *Jon9*9Ci, and *Jon65Aii* (Fig. S10a).

**Fig. 6:**
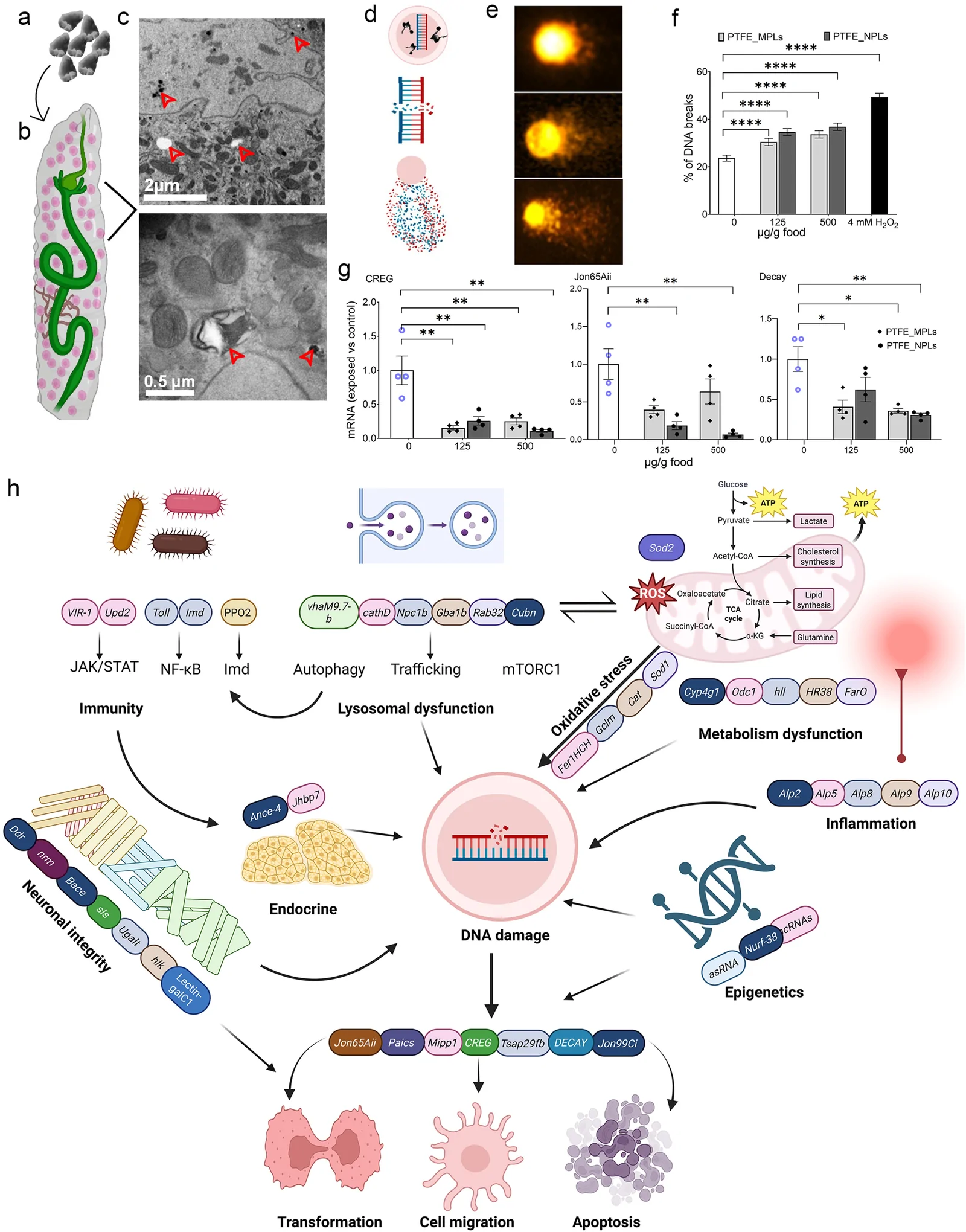
PTFE-MNPLs induce genotoxic effects in Drosophila larvae. (a) Representative PTFE-MNPLs particles, (b) schematic of Drosophila larvae highlighting haemocytes as primary PTFE genotoxicity targets, and (c) TEM images of internalised PTFE particles near the nucleus in larval midgut cells. (d) A schematic illustrates PTFE particle interactions with DNA, leading to strand breaks confirmed by the comet assay. (e) Representative comet assay images show progressively increased DNA fragmentation in partially, moderately, and severely affected haemocytes. (f) Quantitative analysis reveals a concentration-dependent increase in DNA damage, with PTFE-NPLs inducing more severe genotoxic effects. (g) RT–qPCR analysis confirmed transcriptional downregulation of CREG, Jon65Aii, and Decay. (h) Schematic of potential PTFE-MNPL-mediated genotoxic and cellular stress pathways. Data are presented as mean ± SD; statistical significance was determined using two-way ANOVA with Tukey’s multiple comparisons test (∗*p* ≤ 0.05, ∗∗*p* ≤ 0.01, ∗∗∗*p* ≤ 0.001, ∗∗∗∗*p* ≤ 0.0001).

RT-PCR analysis further validated *CREG*, *Jon65Aii*, and *Decay* as representative genes affected by PTFE exposure (Fig. 6g). Collectively, the genotoxic effects of PTFE particles are associated with mitochondrial dysfunction, disrupted redox balance, impaired metabolism, and alterations in autophagy and cytoskeletal pathways, potentially initiating a hazardous cascade (Fig. 6h). These combined stressors, along with ROS accumulation and intracellular particle deposition, create a cellular environment conducive to DNA damage and genomic instability, particularly upon exposure to NPLs.

## 4 Discussion

### 4.1 PTFE-MNPLs traverse the gut, interact with bacteria, partially degrade, and compromise tissue integrity

Although PTFE particles may not immediately compromise organismal viability, they can subtly activate hidden disease pathways that threaten long-term health. Once internalised, these particles initiate complex molecular and physiological responses, revealing how biological systems detect, adapt to, and interact with their presence. PTFE particles, both at micro- and nanoscale, were efficiently internalised into the larval intestine and widely dispersed throughout the midgut lumen. PTFE-MNPLs showed extensive distribution, interacting with symbiotic bacteria, being enclosed within intestinal vesicles, and attaching to the intestinal membrane, including the protective peritrophic chitinous layer. The midgut’s dynamic chemical and mechanical environment markedly altered particle properties, causing significant shrinkage of both MPLs and NPLs. Remarkably, fragmentation of PTFE-MNPLs resulted from increased particle fragility rather than size reduction, releasing nanoscale fragments and highlighting complex mechanisms with potential health implications [20]. *Ex vivo* experiments using larval fluids confirmed these transformations: MPLs shrank by approximately 43% to 1474 nm, forming surface pores averaging about 252 nm, while NPLs decreased by approximately 14% to 214 nm, producing pores averaging about 46 nm. These findings mirror natural biological degradation processes, in which larger plastic particles progressively fragment into nanoscale debris; for instance, rotifers can generate roughly 3.5 × 10^5^ NPLs per day from ingested MPLs [21]. Collectively, these results highlight the continual formation of secondary NPLs and ultra-small plastic species. Among these, oligomers and monomers represent an emerging, largely uncharacterised class of materials whose detection is challenged by their minute size, structural complexity, and the lack of standardized analytical methods [22]. Biological interactions with such particles may induce histological and physiological disturbances, which should be investigated at the cellular and molecular levels to better understand the potential health implications.

### 4.2 Transcriptomic analysis revealed how PTFE-MNPLs disrupt cellular homeostasis, targeting lysosomes and mitochondria

Transcriptomic analysis revealed how MNPLs disrupt cellular homeostasis [23]. Exposure to PTFE-MNPLs induces widespread, concentration- and size-dependent transcriptional changes, impacting metabolism, mitochondrial function, redox balance, immunity, oxidative stress, and development. MNPLs enter the *Drosophila* gut, interact with microbiota and the intestinal barrier, and disseminate through enterocytes, engaging lysosomes and mitochondria before reaching the haemolymph. These findings link molecular changes to tissue- and cell-level disturbances, highlighting systemic effects and disruption of essential biological processes [24]. The present findings provide a comprehensive view of how PTFE-MNPLs elicit multilevel biological disturbances, integrating structural, physiological, and transcriptomic responses. The consistent alteration of a core set of 110 DEGs across particle sizes and concentrations suggests a common and systemic impact of PTFE exposure, further validated by RT–qPCR. The initial accumulation of PTFE-MNPLs within the midgut and their direct interaction with symbiotic microbiota resulted in evident cellular damage, revealing the susceptibility of these microbial communities and their potential role in compromising host immunity and homeostasis [25]. Notably, the dysregulation of immune-related genes such as *LysC, PGRP-SC1a, Upd2, NimB5, VIR-1, IRC, Ag5r2,* and *PPO2* reflects a coordinated yet disrupted immune response in the gut epithelium. Gut microbiota composition is largely regulated by antimicrobial peptides and lysozymes such as *LysC*, linking microbial perturbations to immune activation [26]. *Upd2* activates the JAK/STAT pathway to orchestrate development, immunity, and homeostasis (Table S1). *PGRP-SC1a* mediates pathogen phagocytosis to ensure host survival. Within the gut, *Ag5r2* regulates protease activity and protects against pathogen invasion, while the extracellular *Catalase IRC* safeguards gastrointestinal integrity, its depletion causing severe implications. RT-qPCR validation confirmed that PTFE exposure perturbs key immune regulators, including *Toll*, *Imd*, and *BaraA1*. The NF-κB pathways *Toll* and *Imd* govern most inducible immune effectors essential for survival, while *BaraA1*, strongly induced in the fat body yet expressed systemically, underscores widespread immune disruption. This dysregulation likely reflects PTFE interference with host–microbiota interactions, extending effects beyond immunity. Notably, *VIR-1*, a JAK/STAT-dependent antiviral gene typically unresponsive to general stressors, was downregulated, indicating modulation beyond canonical antiviral pathways. Similarly, *PPO2*, an Imd pathway component, is reduced under hypoxia, revealing disrupted metabolic–immune crosstalk, while *NimB5* links nutrient status to immune regulation, maintaining energy balance and survival under starvation. Notably, PTFE exposure induces immune dysregulation in females, shifting toward a Th2-dominant profile and altering Immunoglobulin G subclass ratios [18].

Beyond their potential effects on the microbiota, our study revealed that PTFE-MNPLs traverse the intestinal barrier via two distinct pathways. The first follows normal intestinal transport through vesicular trafficking, whereas the second represents an unanticipated route, allowing direct penetration into enterocytes. Remarkably, PTFE-NPLs exploited this pathway, entering enterocytes and potentially reaching systemic circulation, highlighting a significant risk of stronger cellular and physiological effects. While MNPL entry generally aligns with established transport mechanisms, as observed for polylactic acid (PLA)-NPLs [27], direct cellular penetration has been reported for metal nanoscale materials, such as TiO_2_ nanoparticles [28]. Importantly, our study identifies a novel and previously uncharacterised pathway for MNPL penetration. Once internalised, PTFE particles trigger complex, coordinated cellular responses. Lysosomes are severely affected, appearing enlarged and vacuolated, while mitochondria exhibit swelling, cristae disruption, vacuolation, and other structural abnormalities, marking them as primary targets of PTFE-induced cellular stress.

Lysosomes act as central hubs of cellular homeostasis, integrating degradation, metabolism, and immune regulation. However, nanoparticle accumulation destabilises their membranes, impairs autophagy, induces oxidative stress, and triggers cell death, establishing lysosomal dysfunction as a hallmark of cellular toxicity [29]. PTFE-MNPL exposure markedly dysregulated several lysosome-associated genes, including core lysosomal components *vhaM9.7-b*, *cathD*, *Npc1b*, and *Gba1b*, and lysosome-associated regulators *Rab32* and *Cubn*. The proton pump *vhaM9.7-b* maintains lysosomal acidity essential for enzymatic degradation, and its dysfunction is associated with disease [30]. The protease *cathD* regulates apoptosis, proliferation, and migration, while *Npc1b* and *Gba1b* govern sterol absorption, midgut maturation, autophagy, and programmed cell death, linking lysosomal function to metabolic and developmental processes. In parallel, *Cubn* supports toxin clearance and endocytic trafficking, and *Rab32* modulates mTORC1 signalling to integrate nutrient and energy cues that regulate growth and autophagy. The morphological and transcriptional alterations associated with these genes indicate lysosomal dysfunction as a potential consequence of PTFE-MNPL exposure, with possible effects on mitochondrial function and metabolic integrity.

Mitochondria and lysosomes are among the most important organelles in cells. They form dynamic inter-organelle contact sites where mitochondria regulate lysosomal dynamics and lysosomes influence mitochondrial fission. These contacts enable inter-organelle metabolite transfer and may contribute to diseases linked to mitochondrial and lysosomal dysfunction [31]. Exposure to PTFE-MNPLs induces mitochondrial swelling, cristae disruption, and vacuolation, accompanied by widespread transcriptional reprogramming, establishing mitochondrial dysfunction as a central outcome that intensifies with increasing particle concentration and decreasing size. As the cellular powerhouses, mitochondria integrate metabolic and bioenergetic pathways, and their impairment destabilises cellular homeostasis [32]. Among the deregulated genes, *Odc1*, *hll, HR38, FarO, Cyp4g1*, and *phu* are metabolism-linked and mitochondria-related: *hll* and *HR38* govern lipid and glycogen metabolism, *FarO* and *Cyp4g1* mediate fatty acid metabolism and PI3K-dependent growth signalling, and *phu*, a hypoxia-responsive gene, signals compromised metabolic resilience. *Odc1* links polyamine metabolism to lipid storage, and reduced *Odc1* in *Drosophila* increases body weight and triglyceride storage by enlarging fat cells and upregulating fatty acid synthesis genes, without affecting food intake [33]. Mitochondrial damage and metabolic disruption following exposure to PTFE-derived MNPLs were associated with the deregulation of several alkaline phosphatases (ALPs), including *Alp2, Alp5, Alp8, Alp9, and Alp10*, as detected by omics analyses and RT-qPCR. ALPs are membrane-bound enzymes that mitigate inflammation and metabolic disorders by detoxifying lipopolysaccharides (LPSs) [34].

### 4.3 Transcriptomic analysis revealed how PTFE-MNPLs increase ROS levels, affect development, and related traits

In addition to their metabolic dysfunction, mitochondria act as a primary source of reactive oxygen species (ROS) during dysregulated ATP production [35]. Correspondingly, PTFE-MNPL exposure induced pronounced oxidative stress, with the highest PTFE-NPL concentration elevating ROS levels more than 4-fold in *Drosophila* haemocytes. S*od2,* a key mitochondrial antioxidant enzyme [36], was downregulated by PTFE-NPLs but unaffected by PTFE-MPLs, revealing size-dependent oxidative stress [37]. Whole-larval transcriptomes showed suppression of respiratory chain complex genes (I–V), indicating disrupted oxidative phosphorylation, impaired ATP production, and consequent energy imbalance, oxidative stress, and apoptosis with PTFE-MNPL exposure [38,39]. Defective OXPHOS, reduced ATP synthesis, and excessive ROS collectively contribute to mitochondrial dysfunction and cell death [40], consistent with observed mitochondrial membrane potential loss across both PTFE particle sizes and concentrations. PTFE-MNPL exposure impairs mitochondrial function and AKT/mTOR signalling in murine intestinal organoids, reducing metabolic activity and inducing cell death [41], while in *Drosophila*, it disrupts energy metabolism, sleep, and lipid and protein homeostasis [42]. Transcriptomic analysis showed that only high-concentration PTFE-NPLs downregulated key antioxidant genes, including *Cu/Zn Sod (Sod1)* and *Cat*, whereas *Gclm* and *Fer1HCH* were downregulated regardless of PTFE concentration or size, persisting especially in NPL-exposed larvae. Loss of *Gclm* impairs glutathione synthesis and disrupts redox balance, while loss of *Fer1HCH* increases ROS, promotes ferroptosis, alters Hippo signalling, disrupts haemocytes differentiation, and causes diverse developmental defects in *Drosophila*. RT-qPCR validation confirmed significant regulation of *Cu/Zn Sod*, *Cat*, and *Mtna*, the latter protecting cells from oxidants and electrophiles that react with sulfhydryl groups [43].

Disruption of metabolic and mitochondrial function, accompanied by impaired redox balance, can profoundly affect development and contribute to diverse pathologies, including endocrine disorders. In *Drosophila*, *Ance-4*, an ACE-like protein with potential regulatory roles in peptide hormone signalling, and *Jhbp7*, a juvenile hormone-binding protein involved in hormone transport during pupation, are both perturbed by PTFE-MNPL exposure. PTFE-MNPLs also disrupt genes critical for neuromuscular and neuronal integrity, including *Bace*, *sls*, *Ugalt*, *hlk*, *nrm*, *Ddr*, and *Lectin-galC1*. These transcriptional alterations may impair amyloid-beta processing, muscle fibre stability, synaptic signalling, and neuronal maintenance, linking PTFE-induced oxidative and metabolic stress to neuromuscular dysfunction and potential neurodegenerative outcomes in *Drosophila*.

PTFE-MNPL exposure disrupts key epigenetic and transcriptional regulators such as *Nurf-38*, essential for nutrient-responsive larval growth [44], and may alter non-coding RNAs (*lncRNAs, asRNA*) that control developmental, neural, and gonadal functions as well as stress responses [45,46]. Furthermore, PTFE-MNPLs affect the salivary gland–specific transcription factor *Sage*, which regulates organ-specific gene networks essential for tissue survival and morphogenesis, demonstrating their capacity to broadly disrupt transcriptional and epigenetic regulatory programs [47]. High-concentration PTFE-NPLs impair reproductive capacity by disrupting sperm flagellum structure, as indicated by ORA of GO pathways. RT–qPCR validation showed that *ms(3)K81*, a paternal-effect gene essential for early embryonic divisions in *Drosophila* [48,49], is downregulated following PTFE-MNPL exposure. Similar downregulation with PS-NPLs supports a mechanism by which PTFE and other MNPLs compromise male reproductive function [50]. Notably, Zhang et al. reported that the reproductive impacts of PTFE-MNPLs are more pronounced than those of other polymer types [51]. The reproductive impacts of PTFE-MNPLs include reduced oocyte maturation in mice and molecular perturbations that may contribute to genotoxicity and carcinogenic outcomes [52].

### 4.4 Transcriptomic analysis revealed how PTFE-MNPLs affect DNA integrity

Our findings demonstrate that PTFE-MNPLs induce concentration- and size-dependent DNA damage in *Drosophila* haemocytes, reflecting profound disruption of cellular homeostasis and widespread dysregulation across multiple pathways. While the genotoxic potential of PTFE-MNPLs remains unreported, smaller particles induce oxidative stress and inflammation in human cells, indicating a potential for genotoxic effects [17]. Extended exposure to MNPLs induces genotoxicity and carcinogenic phenotypes, including anchorage-independent growth and invasiveness, with transcriptomic analyses revealing upregulation of oncogenes and lung cancer-associated genes over time [53]. Our results showed that transcriptomic profiling reveals PTFE exposure perturbs key regulators of apoptosis, cell migration, and tissue integrity, including *Decay*, *Mipp1*, *Tsap29fb*, *CREG*, *Paics*, *Jon9*9Ci, and *Jon65Aii*. *DECAY*, a caspase homolog, mediates programmed cell death, whereas *Mipp1* stabilises filopodia and promotes collective cell migration via inositol phosphate signalling. *Tsap29fb* functions as a tumour suppressor, restraining EGFR signalling and Ras-associated epithelial transformation, while *CREG* regulates proliferation, differentiation, and apoptosis through ERK1/2, PI3K, and JNK pathways. *PAICS* enhances purine synthesis to support cell survival and proliferation. PTFE-MNPL exposure also modulates extracellular matrix-regulating genes, including *Jon9*9Ci and *Jon65Aii*, suggesting compromised cell migration, tissue architecture, polarity, and potentially metastasis formation [54,55]. Collectively, these coordinated transcriptional changes indicate that PTFE-MNPLs undermine apoptosis, tissue integrity, and cellular polarity, potentially engaging pathways linked to genotoxicity and carcinogenesis. Despite the hazards posed by MPLs, NPLs present an even greater risk, as their smaller size enhances bioavailability, fragment diffusion, and biological reactivity, making physical properties a more critical determinant of toxicity [56].

In summary, when extending our results to potential human exposure, using Teflon™ (PTFE) utensils can introduce MNPLs into the body, which penetrate the digestive system and interact with gut bacteria, potentially disrupting microbial balance and compromising digestive and immune functions. Once internalised, these particles impair lysosomal and mitochondrial function, induce oxidative stress, and perturb cellular metabolism. Over time, cumulative effects may impact energy homeostasis as well as reproductive and neuromuscular processes. Consistent molecular and cellular disturbances may contribute to DNA damage, tissue dysfunction, and activation of carcinogenic pathways, suggesting potential long-term health implications of everyday MNPL exposure. While quantitative risk assessment was beyond the scope of this study, future work incorporating exposure modelling and dose–response analyses would help better define these risks. Importantly, our study identified several genes of unknown function that were consistently altered by PTFE exposure, regardless of particle size or concentration. These genes, associated with atypical pathways, may provide valuable clues to PTFE-specific toxicity mechanisms and warrant further investigation. Despite our findings, some limitations should be acknowledged. Human exposure and environmental distribution of MNPLs remain uncertain due to variability in measurement methods, units, and limited sensitivity, particularly for NPLs, highlighting the need for quantitative exposure modelling and dose–response analyses to better inform experimental and diagnostic studies. Higher particle concentrations are often used *in vitro* to reveal biological effects, and future studies should translate these findings to more realistic exposure scenarios. Commercially sourced PTFE particles offer advantages such as high purity, well-defined size, and reproducibility, enabling controlled experimental conditions. However, they differ from particles generated during high-temperature cooking, which are typically thermal degradation products with altered surface chemistry, charge, and co-contaminants. Using particles that better mimic real-life exposure could provide more physiologically relevant insights, particularly given PTFE’s widespread use and limited human health data. Microbial community analyses using 16S rRNA sequencing or metagenomics can reveal early-stage impacts of MNPLs and PTFE, including potential dysbiosis that may directly affect host health, and can further support findings from transmission electron microscopy or associated gene expression changes. MNPLs are subject to chemical, mechanical, and biological interactions, including changes in surface chemistry, which may lead to bio-erosion or fragmentation. Complementary techniques such as gel permeation chromatography, X-ray photoelectron spectroscopy, and transmission or scanning electron microscopy combined with Fourier-transform infrared or Raman spectroscopy can provide detailed insights into particle fate, structural alterations, and surface chemical changes. Finally, mass spectrometry-based metabolomics or proteomics can reveal molecular changes induced by particle exposure, complementing transcriptomic data to provide a comprehensive view of PTFE impacts at the molecular level. This integrated approach can help identify key pathways engaged in the response and explain the observed structural and physiological effects. These approaches provide a comprehensive basis for exploring how PTFE-MNPLs interact with biological systems, affect molecular and physiological processes, and inform future studies in more realistic environmental and health contexts.

## 5 Conclusion

In the *Drosophila* model, oral exposure to PTFE-MNPLs leads to their internalisation within the intestinal lumen. There, they interact with symbiotic bacteria, which cause cellular damage and trigger transcriptional changes related to immune pathways, representing the initial level of impact on exposed individuals. Physicochemical interactions in the midgut are associated with particle fragmentation, potentially releasing ultra-small secondary NPLs, oligomers, and monomers. The intestinal barrier fails to fully prevent PTFE-MNPL translocation, as MPLs are internalised via vesicular transport, while NPLs exploit two distinct routes, penetrating the peritrophic membrane and migrating along vascular vacuoles. Once inside enterocytes, MNPLs distribute widely and interact with key organelles, inducing morphological and physiological disruptions. Lysosomes are enlarged and vacuolated, while mitochondria exhibit swelling, cristae disorganization, vacuolation, and other structural abnormalities, identifying them as primary targets of PTFE-induced cellular stress. These disturbances compromise molecular and cellular homeostasis, affecting metabolism, mitochondrial function, redox balance, immunity, epigenetic and transcriptional regulation, neurodegeneration, apoptosis, intracellular trafficking, development, and potentially carcinogenic pathways. Smaller NPLs exert particularly pronounced effects, destabilising redox balance, impairing antioxidant defence, and promoting DNA damage. Collectively, these findings establish PTFE-MNPLs as dynamic biological disruptors, transforming from environmental pollutants into potentially potent modulators of cellular and organismal homeostasis, with multi-layered impacts on the health of the exposed organisms.

## CRediT authorship contribution statement

**Mohamed Alaraby:** Writing – original draft, Investigation, Conceptualization. **Doaa Abass:** Methodology, Investigation. **Elham Farghal Elkady:** Investigation. **Raquel Egea:** Software, Methodology. **Antonia Velázquez:** Supervision. **Alba Hernández:** Writing – review & editing, Supervision, Funding acquisition, Conceptualization. **Ricard Marcos:** Writing – review & editing, Supervision, Conceptualization.

## Data availability statement

RNA-seq data deposited in GEO under accession GSE324731(link: https://www.ncbi.nlm.nih.gov/geo/query/acc.cgi?acc&equals;GSE324731). Other data available upon request from the corresponding author.

## Declaration of competing interest

The authors declare no competing interests.
