# Supplementary material for: Polytetrafluoroethylene utensils as a source of micro- and nanoplastics: Insights from a Drosophila model
Source: Eco Environ Health. 2026 Sep 15;5(4):100285. doi: 10.1016/j.eehl.2026.100285 (PMC13653986; doi:10.1016/j.eehl.2026.100285)
Supplement: Supplementary file 1 [file mmc1.docx]

**Supplementary Material**

**Polytetrafluoroethylene utensils as a source of micro- and nanoplastics: Insights from a *Drosophila* model**

Mohamed Alaraby^a,b,^*, Doaa Abass^b^, Elham Farghal Elkady^c^, Raquel Egea^a^, Antonia Velázquez^a^, Alba Hernández^a^, Ricard Marcos^a,^*

^a^Group of Mutagenesis, Department of Genetics and Microbiology, Faculty of Biosciences, Universitat Autònoma de Barcelona, 08193 Cerdanyola del Vallès, Spain.

^b^ Zoology Department, Faculty of Sciences, Sohag University, Sohag 82524, Egypt.

^c^ Plant Protection, Institute of Agricultural Research Center, Sohag 82524, Egypt.

*Corresponding authors.

Email: ricard.marcos@uab.cat (R. Marcos); mohamed.alaraby@uab.cat (M. Alaraby)

2186.73 ± 1372.11 nm

e

h

g

249.41 ± 60.12 nm

f

j

PTFE_MPLs

k

PTFE_NPLs

l

i

a

b

c

d

**Fig. S1.** Structural, morphological, and chemical characterisation of PTFE-MNPLs. Representative SEM (a,c) and TEM (b,d) images of PTFE-MPLs (a,b) and PTFE-NPLs (c,d). Size distribution histograms derived from ImageJ analysis of PTFE-MPLs (e) and PTFE-NPLs (f). (g) Dynamic light scattering (DLS) measurements. (h) Zeta (ζ)-potential value. Fourier-transform infrared (FTIR) spectra of PTFE-MPLs (i) and PTFE-NPLs (j). Energy-dispersive X-ray spectroscopy (EDX) of PTFE-MPLs (k) and PTFE-NPLs (l).

a

b

a

b

**Fig. S2. Viability of *Drosophila melanogaster* following exposure to graded concentrations of PTFE–MNPLs. a) Scheme of the *D. melanogaster* life cycle, showing experimental exposure to PTFE particles incorporated into standard food media.** Eggs (n = 50 per replicate, five replicates) were placed on food containing PTFE particles at 31.25, 125, and 500 µg/g and allowed to develop through larval and pupal stages. **b**) Adult emergence was measured, showing that PTFE particle exposure did not significantly affect viability compared with controls. Statistical analysis was conducted using one-way ANOVA with Dunnett’s multiple comparisons test (significance: *p* ≤ 0.05).

**Fig. S3. TEM images showing internalisation of PTFE–MPLs and PTFE–NPLs in intestinal bacteria of exposed larvae. (a–d**) Localisation and uptake of PTFE–MPLs in the larval midgut. **a**) PTFE–MPLs dispersed in the midgut lumen near symbiotic bacteria. **b**) PTFE–MPLs attached to the bacterial surface. **c**) Internalisation of PTFE–MPLs into bacterial cells. **d**) Structural damage to bacteria associated with PTFE–MPL exposure. **e–h**) Localization and uptake of PTFE–NPLs in the larval midgut. **e**) PTFE–NPLs dispersed in the midgut lumen near symbiotic bacteria. **f**) PTFE–NPLs adhered to the bacterial surface. **g**) Internalisation of PTFE–NPLs into bacterial cells. **h**) Bacterial damage associated with PTFE–NPL exposure. Orange arrows indicate PTFE–MPLs, yellow arrows indicate PTFE–NPLs, and red arrows indicate bacterial cells (intact or damaged).

**Fig. S4.** TEM analysis reveals surface degradation of PTFE–MPLs following internalisation in exposed larvae. **a**) Intact PTFE–MPLs with smooth, undamaged surfaces observed in dispersion solution. **b**) TEM image of the intestinal lumen of an unexposed larva showing the absence of PTFE–MPLs. **c–e**) PTFE–MPLs within the intestinal lumen of exposed larvae exhibit surface deterioration, including visible pores and structural irregularities, suggesting degradation during or after internalisation. **f–h**) Threshold-adjusted images of panels **c–e** highlighting pore formation and the release of fragments from the particle surface. Red arrows point to detached fragments or pore-like surface features associated with particle erosion.

**Fig. S5.** Assimilation and biodegradation of PTFE–MPLs in larval fluids. To investigate the biological degradation of PTFE–MPLs, microparticles were incubated for 72 h under gentle agitation in either phosphate-buffered saline (PBS) or in gut fluids extracted from third instar *Drosophila* larvae. **a**) TEM imaging of the PTFE–MPL dispersion prior to incubation shows intact particles with smooth, undamaged surfaces. **b**) after exposure to larval fluids, microparticles exhibited notable surface degradation, including pore formation, which became more pronounced in higher-magnification images (**c, d**). **e**) some particles were observed within bacterial cells present in the larval fluid, suggesting microbial internalisation and possible involvement in the degradation process. **f**) Continued interaction with both the intestinal fluids and bacteria led to structural breakdown of the PTFE–MPLs, resulting in the formation of smaller fragments (**g**). Elemental analysis using TEM–EDX confirmed the presence of fluorine in PTFE–MPLs located in larval fluids (**h**) and inside bacteria (**i**), consistent with the chemical signature of PTFE. The median and interquartile range (IQR) of PTFE–MPLs diameter after incubation in PBS (2346.5 and 2131.0) compared to larval fluids (572.7 and 2181.6), indicating biological erosion (**j**). **k**) illustrates the emergence of smaller PTFE particles resulting from degradation (251.52 ± 84.83 nm), further supporting the role of larval fluids and associated microbiota in the breakdown of PTFE–MPLs. Red arrows indicate to PTFE–MPLs. Statistical analysis was performed using a Wilcoxon test *(****p* ≤ *0.0001*).

**Fig. S6.** Assimilation biodegradation of PTFE–NPLs in larval fluids. PTFE–NPLs were incubated for 72 h under gentle agitation in either PBS or gut fluid extracts obtained from third instar *Drosophila* larvae. **a**) TEM imaging of PTFE–NPLs in suspension revealed well-dispersed nanoparticles with intact, smooth surfaces. Upon incubation with larval fluids, PTFE–NPLs were observed attaching to gut-associated bacteria (**b**), and several particles were identified within bacterial cells (**c**), suggesting microbial internalisation and a potential role in nanoplastic degradation. Extended exposure to the larval microenvironment resulted in significant structural alterations to the nanoparticles, including pore formation and surface erosion (**d**). Quantitative particle size analysis (**e**) revealed a significant decrease in average diameter following incubation in larval fluids (214.10 ± 53.98 nm) compared to PBS controls (249.85 ± 61.93 nm), consistent with biologically mediated degradation. **f**) Measurements of surface pore diameters showed a mean size of 46.0 ± 13.97 nm. Elemental analysis via TEM–EDX confirmed the presence of fluorine in PTFE–NPLs located both in the extracellular matrix of the larval fluids (**g**) and within bacterial compartments (**h**), with corresponding spectral data shown in (**i**) and (**j**), respectively. These findings confirm the chemical identity of PTFE nanoparticles in both the extracellular fluid and within bacterial cells, respectively. Red arrows indicate to PTFE–NPLs. Statistical analysis was performed using a paired t-test (***p* ≤ *0.01*).

**Fig. S7. TEM analysis reveals stepwise internalisation of PTFE–MPLs and PTFE–NPLs in midgut. Transmission electron microscopy was used to visualize the uptake pathway of micro- and nanoscale PTFE particles in exposed larvae. For PTFE–MPLs, particles are initially detected in the midgut lumen surrounded by lysosome-like vesicles (a), followed by accumulation near or within the peritrophic membrane adjacent to the microvilli (b). The particles are subsequently observed crossing the microvillar barrier and entering the cytoplasm of enterocytes (c). A similar internalisation route is observed for PTFE–NPLs: particles localise in the lumen in proximity to lysosomal vesicles (d), interact with the peritrophic membrane and vesicular structures at the epithelial interface (e), become enclosed within intracellular vesicles near the apical membrane (f). These findings indicate that both PTFE–MPLs and PTFE–NPLs follow a vesicle–mediated internalisation process that culminates in lysosomal digestion within gut epithelial cells.** Orange arrows indicate PTFE–MPLs and yellow arrows PTFE–NPLs.

**Fig. S8.** TEM analysis reveals intracellular localization and mitochondrial interactions of PTFE–MPLs and PTFE–NPLs in larval midgut cells. **a–d**) PTFE–MPLs are localised within the cytoplasm of enterocytes, where they interact with mitochondria and lysosomal vesicles. **a**) PTFE–MPLs are observed adjacent to mitochondria. **b**) particle morphology is altered within lysosomal compartments, accompanied by changes in mitochondrial structure and increased electron density, indicative of mitochondrial stress. **c,d**) mitochondrial damage is evident, potentially resulting from direct PTFE–MPL internalisation. **e–h**) PTFE–NPLs follow a similar intracellular pathway. **e**) particles are seen translocating through microvilli into the cytoplasm, where electron-dense vesicles accumulate near mitochondria. **f**) PTFE–NPLs localise close to mitochondria, and in (**g**), they are found within mitochondria, suggesting direct organelle invasion. **h**) mitochondrial structural disruption is apparent, likely due to widespread lysosomal vesicle accumulation and direct PTFE–NPL contact. Orange arrows indicate PTFE–MPLs, yellow arrows indicate PTFE–NPLs, and red arrows mark mitochondria (intact or damaged).

**Fig. S9.** Transcriptomic responses of larvae to PTFE–MPLs and PTFE–NPLs exposure. **a**) Venn diagram showing DEGs unique to or shared between PTFE–MPL- and PTFE–NPL-exposed samples. **b**) Numbers of DEGs in each treatment group (158, 553, 975, and 2,897 for MPL_125, MPL_500, NPL_125, and NPL_500, respectively), demonstrating a concentration- and particle size–dependent transcriptomic response. **c**) GSEA reveals distinct enrichment profiles for MPL and NPL exposure, with significant associations to curated biological pathways. **d**) ORA identifies key functional categories and biological processes affected by PTFE exposure. **e**) GSEA of GO Cellular Component terms shows PTFE–MPL and PTFE–NPL deregulation of specific cellular pathways. **f**) ORA bubble plot illustrating GO Biological Process pathways enrichment across treatments, where bubble size indicates gene count and colour denotes q-value significance. **g**) KEGG pathway GSEA reveals downregulation of pathways related to xenobiotic metabolism (cytochrome P450), antioxidant and metabolic defence (glutathione, thiamine), RNA processing, lysosomal function, and innate immune signalling (*Toll, Imd, TLR*). HD – human disease.

f

c

b

g

e

a

d

**Fig. S10.** Heat map of 110 common DEGs in *Drosophila* larvae exposed to PTFE–MPLs and PTFE–NPLs at 125 and 500 µg/g food. Genes consistently differentially expressed across all treatment conditions, showing uniform expression patterns independent of particle size or concentration; this subset includes genes with well-characterised biological functions (**a**) and genes with uncharacterised or unknown functions (**b**).

a

b

**Fig. S11.** Transcriptomic analysis pipeline diagram. The pipeline depicts the flow of the transcriptomic data performed with R. The green parallelograms indicate input/output files, and each square represents an analysis step. The name of each package used is included in the lilac color square.

**Table S1**. References not included in the main text referring to characteristics of the genes of interest.

| **Gene** | **Reference** |
| --- | --- |
| *Upd2* | J.C.G. Hombría, S. Brown, S. Häder, M.P. Zeidler, Characterisation of Upd2, a *Drosophila* JAK/STAT pathway ligand. Develop. Biol. 288 (2005) 420–433, https://doi.org/10.1016/j.ydbio.2005.09.040. |
| *PGRP-SC1a* | L.S. Garver, J. Wu, L.P. Wu, The peptidoglycan recognition protein PGRP-SC1a is essential for Toll signaling and phagocytosis of *Staphylococcus aureus* in *Drosophila*. Proc. Nat. Acad. Sci. 103 (2006) 660–665, https://doi.org/10.1073/pnas.0506182103. |
| *Ag5r2* | T. Megraw, T.C. Kaufman, G.E. Kovalick, Sequence and expression of *Drosophila* Antigen 5-related 2, a new member of the CAP gene family. Gene 222 (1998) 297–304, https://doi.org/10.1016/S0378-1119(98)00489-2. |
| *Catalase* *IRC* | E.M. Ha, C.T. Oh, J.H. Ryu, Y.S. Bae, S.W. Kang, I.H. Jang, et al., An antioxidant system required for host protection against gut infection in *Drosophila.* Dev. Cell 8 (2005) 125–132, <https://doi.org/10.1016/j.devcel.2004.11.007>. |
| *Toll* and *Imd* | T. Tanji, Y.T. Ip, Regulators of the Toll and Imd pathways in the *Drosophila* innate immune response. Trends Immunol. 26 (2005) 193–198, https://doi.org/10.1016/j.it.2005.02.006.). |
| *BaraA* | M.A. Hanson, L.B. Cohen, A. Marra, I. Iatsenko, S.A. Wasserman, B. Lemaitre, The *Drosophila* Baramicin polypeptide gene protects against fungal infection. PLoS Pathol. 17 (2021) e1009846, https://doi.org/10.1371/journal.ppat.1009846. |
| *VIR-1* | C. Dostert, E. Jouanguy, P. Irving, L. Troxler, D. Galiana-Arnoux, C. Hetru, et al., The Jak-STAT signaling pathway is required but not sufficient for the antiviral response of *Drosophila.* Nat. Immunol. 6 (2005) 946–953, https://doi.org/10.1038/ni1237. |
| *PPO2* | S. Yu, F. Luo, Y. Xu, Y. Zhang, L.H. Jin, *Drosophila* innate immunity involves multiple signaling pathways and coordinated communication between different tissues. Front. Immunol. 13 (2022) 905370, https://doi.org/10.3389/fimmu.2022.905370.  M. Shin, E. Chang, D. Lee, N. Kim, B. Cho, N. Cha, et al., *Drosophila* immune cells transport oxygen through PPO2 protein phase transition. Nature 631 (2024) 350–359, https://doi.org/10.1038/s41586-024-07583-x. |
| *NimB5* | E. Ramond, B. Petrignani, J.P. Dudzic, J.P. Boquete, M. Poidevin, S. Kondo, et al., The adipokine NimrodB5 regulates peripheral hematopoiesis in *Drosophila.* FEBS J. 287 (2020) 3399–3426, https://doi.org/10.1111/febs.15237. |
| *cathD* | T. Zhang, D. Cheng, C. Wu, X. Wang, Q. Ke, H. Lou, et al., Lysosomal hydrolase cathepsin D non-proteolytically modulates dendritic morphology in *Drosophila.* Neurosci. Bull. 36 (2020) 1147–1157, https://doi.org/10.1007/s12264-020-00479-6. |
| *Npc1b* | Z. Zhou, Z. Yao, M. Abouzaid, J.J. Hull, W. Ma, H. Hua, Y. Lin, Co-expression network analysis: a future approach for pest control target discovery. J. Agric. Food Chem. 71 (2023) 7201–7209, https://doi.org/10.1021/acs.jafc.3c00113. |
| *Gba1b* | Atilano, M.L., Hull, A., Romila, C.A., Adams, M.L., Wildfire, J., Ureña, E., Dyson, M., Ivan-Castillo-Quan, J., Partridge, L. and Kinghorn, K.J., 2023. Autophagic dysfunction and gut microbiota dysbiosis cause chronic immune activation in a Drosophila model of Gaucher disease. Plos Genetics, 19(12), 1011063. https://doi.org/10.1371/journal.pgen.1011063 |
| *Cubn* | A. Atienza-Manuel, V. Castillo-Mancho, S. De Renzis, J. Culi, M. Ruiz-Gómez, Endocytosis mediated by an atypical CUBAM complex modulates slit diaphragm dynamics in nephrocytes. Development 148 (2021) dev199894, https://doi.org/10.1242/dev.199894. |
| *Rab32* | K. Drizyte-Miller, J. Chen, H. Cao, M.B. Schott, M.A. McNiven, The small GTPase Rab32 resides on lysosomes to regulate mTORC1 signaling. J. Cell Sci. 133 (2020) jcs236661, https://doi.org/10.1242/jcs.236661. |
| *hll* and *HR38* | M.S. Thimgan, L. Seugnet, J. Turk, P.J. Shaw, Identification of genes associated with resilience/vulnerability to sleep deprivation and starvation in *Drosophila.* Sleep 38 (2015) 801–814, <https://doi.org/10.5665/sleep.4680>.  A.F. Ruaud, G. Lam, C.S. Thummel, The *Drosophila* NR4A nuclear receptor DHR38 regulates carbohydrate metabolism and glycogen storage. Mol. Endocrinol. 25 (2011) 83–91, https://doi.org/10.1210/me.2010-0337. |
| *FarO* and *Cyp4g1* | E. Cinnamon, R. Makki, A. Sawala, L.P. Wickenberg, G.J. Blomquist, C. Tittiger, et al., *Drosophila* Spidey/Kar regulates oenocyte growth via PI3-kinase signaling. PLoS Genet. 12 (2016) e1006154, <https://doi.org/10.1371/journal.pgen.1006154>.  M. Kefi, P. Konstantinos, V. Balabanidou, C. Sarafoglou, D. Tsakireli, V. Douris, et al., Insights into unique features of *Drosophila* CYP4G enzymes. Insect Biochem. Mol. Biol. 164 (2024) 104041, https://doi.org/10.1016/j.ibmb.2023.104041. |
| *phu* | J. Morimoto, D. Derous, M. Wenzel, Y. Henry, H. Colinet, The transcriptomic signature of physiological trade-offs caused by larval overcrowding in *Drosophila melanogaster.* bioRxiv (2022) 2022-01, https://doi.org/10.1101/2022.01.07.475433. |
| *Gclm* | J. Lim, B.N. Nakamura, I. Mohar, T.J. Kavanagh, U. Luderer, Glutamate cysteine ligase modifier subunit (Gclm) null mice have increased ovarian oxidative stress and accelerated age-related ovarian failure. Endocrinology 156 (2015) 3329–3343, https://doi.org/10.1210/en.2015-1206. |
| *Fer1HCH* | S. Mumbauer, J. Pascual, I. Kolotuev, F. Hamaratoglu, Ferritin heavy chain protects the developing wing from reactive oxygen species and ferroptosis. PLoS Genet. 15 (2019) e1008396, https://doi.org/10.1371/journal.pgen.1008396. |
| *Ance-4*, and *Jhbp7* | X. Houard, T.A. Williams, A. Michaud, P. Dani, R.E. Isaac, A.D. Shirras, et al., The *Drosophila melanogaster*-related angiotensin-I-converting enzymes Acer and Ance: Distinct enzymic characteristics and alternative expression during pupal development. Eur. J. Biochem. 257 (1998) 599–606, https://doi.org/10.1046/j.1432-1327.1998.2570599.x.  S.W. Shin, J.H. Jeon, J.A. Kim, D.S. Park, Y.J. Shin, H.W. Oh, Inducible expression of several *Drosophila melanogaster* genes encoding juvenile hormone binding proteins by a plant diterpene secondary metabolite, methyl lucidone. Insects 13 (2022) 420, https://doi.org/10.3390/insects13050420. |
| *Bace* | S. Singh, V. Kushwaha, S. Sisodia, S. Kumar, K.K. Sahu, Beta-site APP-cleaving Enzyme-1 inhibitory role of natural flavonoids in the treatment of Alzheimer's disease. Cent. Nerv. Syst. Agents Med. Chem. 25 (2025) 39–48, https://doi.org/10.2174/0118715249315049240710063455. |
| *sls* | A.H. Michael, T.A. Hana, V.G. Mousa, K.G. Ormerod, Muscle-fiber specific genetic manipulation of *Drosophila sallimus* severely impacts neuromuscular development, morphology, and physiology. Front. Physiol. 15 (2024) 1429317, https://doi.org/10.3389/fphys.2024.1429317. |
| *Ugalt* | K. Itoh, M. Kurogochi, T. Kaname, J.I. Furukawa, S. Nishihara, Neuromuscular defects in a *Drosophila* model of the congenital disorder of glycosylation SLC35A2-CDG. Biomolecules 15 (2025) 1256, https://doi.org/10.3390/biom15091256. |
| *hlk* | A.D. Elliott, A. Berndt, M. Houpert, S. Roy, R.L. Scott, C.C. Chow, et al., Pupal behavior emerges from unstructured muscle activity in response to neuromodulation in *Drosophila*. Elife 10 (2021) e68656, https://doi.org/10.7554/eLife.68656. |
| *nrm* | A. Kania, H.J. Bellen, Mutations in neuromusculin, a gene encoding a cell adhesion molecule, cause nervous system defects. Roux Arch. Dev. Biol. 204 (1995) 259–270, https://doi.org/10.1007/BF00208493. |
| *Ddr* | M.M. Corty, A.L. Hulegaard, J.Q. Hill, A.E. Sheehan, S.A. Aicher, M.R. Freeman, Discoidin domain receptor regulates ensheathment, survival and caliber of peripheral axons. Development 149 (2022) dev200636, https://doi.org/10.1242/dev.200636. |
| *Lectin-galC1* | M. Bhimreddy, E. Rushton, D.L. Kopke, K. Broadie, Secreted C-type lectin regulation of neuromuscular junction synaptic vesicle dynamics modulates coordinated movement. J. Cell Sci. 134 (2021) jcs257592, https://doi.org/10.1242/jcs.257592. |
| *Decay* | L. Dorstyn, S.H. Read, L.M. Quinn, H. Richardson, S. Kumar, DECAY, a novel *Drosophila* caspase related to mammalian caspase-3 and caspase-7. J. Biol. Chem. 274 (1999) 30778–30783, https://doi.org/10.1074/jbc.274.43.30778. |
| *Mipp1* | Y.L. Cheng, D.J. Andrew, Extracellular Mipp1 activity confers migratory advantage to epithelial cells during collective migration. Cell Rep. 13 (2015) 2174–2188, https://doi.org/10.1016/j.celrep.2015.10.071. |
| *Tsap29fb* | T. Zoranovic, J. Manent, L. Willoughby, R. Matos de Simoes, J.E. La Marca, S. Golenkina, et al., A genome-wide *Drosophila* epithelial tumorigenesis screen identifies Tetraspanin 29Fb as an evolutionarily conserved suppressor of Ras-driven cancer. PLoS Genet. 14 (2018) e1007688, https://doi.org/10.1371/journal.pgen.1007688. |
| *CREG* | E. Kowalewski-Nimmerfall, P. Schähs, D. Maresch, D. Rendic, H. Krämer, L. Mach, *Drosophila melanogaster* cellular repressor of E1A-stimulated genes is a lysosomal protein essential for fly development. Biochim. Biophys. Acta Mol. Cell Res. 1843 (2014) 2900–2912, https://doi.org/10.1016/j.bbamcr.2014.08.012. |
| *Paics* | M.C. Chou, Y.H. Wang, F.Y. Chen, C.Y. Kung, K.P. Wu, J.C. Kuo, et al., PAICS ubiquitination recruits UBAP2 to trigger phase separation for purinosome assembly. Mol. Cell 83 (2023) 4123–4140, https://doi.org/10.1016/j.molcel.2023.09.028. |

**Table S2**. Gene primers of *D. melanogaster* used in quantitative RT-PCR. Primers were design according to National Center for Biotechnology Information NCBI (<https://www.ncbi.nlm.nih.gov/>).

| **No.** | **Gene** | **Forward Primer (5′-3′)** | **Reverse Primer (5′-3′)** | **Function** |
| --- | --- | --- | --- | --- |
| **1** | Transcript variant B (Act5C) **NM_078497.4** | GATGAAGATCCTGACCGAGC | TGTAGAAGGTGTGGTGCCAG | Housekeeping actine 5C (Act5C), as a reference gene, was used for gene normalization. Supports cytoskeleton structure by polymerising actin filaments, essential for cell shape, movement, and division. |
| **2** | Heat shock protein 70 Aa (Hsp70Aa) **NM_169441.2** | CCATCGAGATTGACGCACTG | CGTATGCAACTGCCTCGTCT | Facilitates protein folding and protects cells from stress by binding damaged proteins, involved in heat shock response and hypoxia adaptation. |
| **3** | Heat shock protein 60A (Hsp60A) **NM_167266.1** | ACCAGATCGAGGACACCACC | CGCATCGTAGCCGTAGTCAC | Assists protein folding and protects cells from stress by acting as a molecular chaperone, mainly in mitochondria. |
| **4** | Metallothionein A (Mtna) **NM_001275509.1** | CAATATGCGTCGTGGAAATG | CTAACGCCCGCAACTGTATT | Binds heavy metals to detoxify cells and protect against oxidative stress. |
| **5** | Catalase (Cat) **NM_080483.3** | GAGCCATGAATGTGACGGAC | GTTCTGCACCAGACGCTTCT | Breaks down hydrogen peroxide into water and oxygen, protecting cells from oxidative damage and supporting stem cell balance and signalling. |
| **6** | Superoxide dismutase 2 (Mn) (Sod2) **NM_001299574.1** | ATCGAGTCGCAGTGGAAGAG | GATGCAGCTCCATGATCTCC | Converts mitochondrial superoxide radicals into less harmful molecules, reduces oxidative stress and maintaining mitochondrial function. |
| **7** | Superoxide dismutase 1 (Cu/Zn SOD) **NM_057387.5** | GAAGGTCTCCGGTGAGGTGT | GCGTAATCTTGGAG TCGGTG | Neutralises superoxide radicals in peroxisomes, protecting cells from oxidative damage and supporting lifespan and synaptic plasticity. |
| **8** | Prophenoloxidase 2 (PPO2) **NM_136599.4** | GGCATTGGCATCTGGTCTAC | TCAACCTGGTGCTCTCGAAG | Catalyses melanisation and produces reactive oxygen species to defend against pathogens. |
| **9** | Immune deficiency (Imd) **NM_001299643.1** | TCGATGTGGTTTCCACGCACTT | TCCAGTGCCTTCCAAACCAGTTTC | Regulates innate immune responses and triggers antimicrobial peptide production against bacterial infections. |
| **10** | Toll (toll, transcript variant B (Toll)  **NM_079794.3** | ATCCTCTGGTTTATTCAGCTGGT | TATGGCAGTTGATTACCAGTGCT | Activates innate immune signalling pathways to defend against pathogens and regulate development. |
| **11** | Cytochrome P450 (18a1 cyp18a1) **NM_078679.3** | CGCTGAACGGATATGGCATTATC | CTCATCATCAGGCTGCAGATCA | Metabolises toxins and hormones, contributing to detoxification and developmental processes. |
| **12** | Male sterile (3) K81 (ms (3)K81) **NM_143253.2** | CGCCAACGTATACTAGCCCC | ACCGCAGCTCCGAGTTTATC | Ensures accurate chromosome segregation during sperm development, preventing DNA damage and male sterility. |
| **13** | Alkaline phosphatase 5 (Alp5) **NM_142674** | GCAAGATGTTCCGCCTGTTG | GGTGGTCACACCCATTCCAT | Hydrolyses phosphate groups from molecules, contributing to phosphate metabolism and cellular regulation. |
| **14** | Alkaline phosphatase 10 (Alp10) **NM_139719.2** | CGTCGCCGTACCCATTATGA | TCAGCCAGATTGGGAGCAAG | Hydrolyses phosphate groups from various substrates, playing a role in phosphate metabolism and developmental processes. |
| **15** | alkaline phosphatase 2 (Alp2) **NM_137810.1** | AGTCCACGGGATTTGTGACC | CCCGTGACTATCCTTCGTGG | Removes phosphate groups from molecules, contributing to phosphate metabolism and cellular signalling during development. |
| **16** | Ance-4, transcript variant A (Ance-4) **NM_136598.3** | GTTCGAGCAGATCCGGTGTT | CTGCAGTTGCGCAGCCAAT | Functions as a metallopeptidase involved in peptide processing and regulation, contributing to developmental and physiological processes. |
| **17** | BaraA1, transcript variant A (BaraA1) **NM_206108.2** | AGCTTCCCACACCATCAGTG | ACGCTTCCAAACGGAGACAT | Encodes an antimicrobial peptide involved in innate immune defence and infection response. |
| **18** | Cathepsin D (cathD), transcript variant A **NM_143756.3** | AGCCACCAGAGAAGCGTAAC | TGCCGTTCTTGGTGTAGGTC | Acts as a lysosomal aspartic protease involved in protein degradation, apoptosis, and tissue remodelling; indirectly linked to oxidative stress and mitochondrial function through roles in cell death pathways. |
| **19** | Mayo (CG11318) | CCAGGGAGTGCAGAGTGAAG | ATTCCCACCTCTCTGTCGGA | Encodes a protein involved in immune response and possibly stress adaptation |
| **20** | Juvenile hormone binding protein 7 (Jhbp7) (CG11852) **NM_143100.2** | AGCTCAACTTCAGGGACGTG | ATGGCCACATGAATGGAGGG | Binds juvenile hormone to regulate its stability and transport during development and reproduction. |
| **21** | Uncharacterized protein (CG9993) **NM_137674.2** | CCGGTAGTCAGTTGCTGGAG | CACCTTGGGCTTCGTGATCT | Function currently unknown. |
| **22** | Cellular repressor of E1A-stimulated genes, (CREG) **NM_001260223.1** | AGTCCAGATCCTCCAGCCAT | CAGTCGGGACCAGTGAAGTC | Regulates gene expression by repressing E1A-activated transcription, involved in cell growth and differentiation. |
| **23** | Cubilin (Cubn) **NM_167193.2** | TGTCCTCAAGTTCGTGTCGG | TCGCCGAATCCGTATGAAGG | Functions as an endocytic receptor involved in nutrient absorption and protein reabsorption. |
| **24** | Cytochrome P450 4g1 (Cyp4g1) **NM_080292.4** | TCACGTGCGATTGTTGAGGA | GAAACTAGATCCCGCCGAGG | Metabolises lipids and xenobiotics, contributing to detoxification and regulation of oxidative stress. |
| **25** | Death executioner caspase related to Apopain/Yama (Decay)  **NM_058114.4** | GATCATAATCTCGCGCCCCA | CAGATCGTCGAAGGTGCGTA | Mediates apoptosis by cleaving cellular proteins during programmed cell death, indirectly linked to mitochondrial pathways and cellular stress responses. |
| **26** | Fatty acyl-CoA reductase in oenocytes (FarO) **NM_143764.3** | CTGAATGTCGGAGGCACCTT | GTCCTCCACGGCAAAGAGAA | Catalyses the reduction of fatty acyl-CoA to fatty alcohols, involved in lipid metabolism and cuticle formation. |
| **27** | Heimdall (hll) **NM_135852.4** | GTCAAAGAGGATGGCTGGCT | AGAGCGGCAGTATCATTGGG | Involved in immune response regulation and stress adaptation. |
| **28** | Immune-regulated catalase (Irc) **M_001275724.1** | AGGTCCGATAGAGTCGCAGT | CCTCCAGTCGCTTTTGCCTA | Breaks down hydrogen peroxide to protect cells from oxidative damage, playing a key role in oxidative stress defence. |
| **29** | Jonah 65Aii (Jon65Aii) **NM_139757.2** | ACAACAGTGTCTGCCTGGAC | GGTCAATCCTTTGGGGCTGT | Functions as a digestive serine protease involved in protein breakdown during digestion. |
| **30** | Galactose-specific C-type lectin (Lectin-galC1) **NM_057852.3** | CACTGCATCCACCTTGGCTA | AGTATTCCGGTGGTTCCCCT | Binds specific sugars to mediate immune recognition and pathogen binding |

**Flow cytometry gating strategy and representative plots**

Flow cytometry was conducted using a sequential gating strategy to accurately identify and quantify haemocytes. All events were first visualized using forward scatter (FSC) and side scatter (SSC) to exclude debris and non-cellular particles, defining the primary population (P1). Singlet discrimination (FSC-A vs FSC-H) removed doublets and aggregates, generating a refined population (P2). Where applicable, dead cells were excluded using viability staining. The final gated population (P3) represents biologically relevant haemocytes and was used for quantitative fluorescence analysis of TMRM (Y585-PE-A) and FITC (B525-FITC-A). Background fluorescence was determined using an unstained control and subtracted from all treated and negative control samples to ensure probe-spdecific measurements.

Representative flow cytometry plots, including gating steps and fluorescence distributions, are shown in Figure 5g, illustrating the exclusion of debris and selection of the target haemocyte population. Fluorescence intensity in P3 was quantified as mean fluorescence intensity (MFI), with higher TMRM MFI reflecting intact mitochondrial membrane potential and healthier mitochondria, while lower MFI indicates mitochondrial depolarization or damage.
